# A Body Conformal Ultrasound Receiver for Efficient and Stable Wireless Power Transfer in Deep Percutaneous Charging

**DOI:** 10.1002/adma.202419264

**Published:** 2025-03-26

**Authors:** Iman M. Imani, Hyun Soo Kim, Minhyuk Lee, Seung‐Bum Kim, So‐Min Song, Dong‐Gyu Lee, Joon‐Ha Hwang, Jeyeon Lee, In‐Yong Suh, Sang‐Woo Kim, Jun Chen, Heemin Kang, Donghee Son, Jeong Min Baik, Sunghoon Hur, Hyun‐Cheol Song

**Affiliations:** ^1^ Electronic and Hybrid Materials Research Center Korea Institute of Science and Technology (KIST) Seoul 02792 Republic of Korea; ^2^ Department of Materials Science and Engineering Korea University Seoul 02841 Republic of Korea; ^3^ School of Mechanical Engineering Korea University Seoul 02841 Republic of Korea; ^4^ School of Advanced Materials Science and Engineering Sungkyunkwan University (SKKU) Suwon 16419 Republic of Korea; ^5^ Department of Micro/Nano Systems Korea University Seoul 02841 Republic of Korea; ^6^ Department of Materials Science and Engineering Center for Human‐oriented Triboelectric Energy Harvesting Yonsei University Seoul 03722 Republic of Korea; ^7^ Department of Bioengineering University of California Los Angeles CA 90095 USA; ^8^ Department of Electrical and Computer Engineering Sungkyunkwan University (SKKU) Suwon Republic of Korea; ^9^ Department of Superintelligence Engineering Sungkyunkwan University (SKKU) Suwon Republic of Korea; ^10^ Center for Neuroscience Imaging Research Institute for Basic Science (IBS) Suwon Republic of Korea; ^11^ KIST‐SKKU Carbon‐Neutral Research Center Sungkyunkwan University (SKKU) Suwon 16419 Republic of Korea

**Keywords:** ferroelectric and dielectric booster, flexible implantable energy harvester, rechargeable deep‐tissue implantable devices, ultrasound‐driven triboelectric nanogenerator, wireless battery charging

## Abstract

Wireless powering of rechargeable‐implantable medical devices presents a challenge in developing reliable wireless energy transfer systems that meet medical safety and standards. Ultrasound‐driven triboelectric nanogenerators (US‐TENG) are investigated for various medical applications, including noninvasive percutaneous wireless battery powering to reduce the need for multiple surgeries for battery replacement. However, these devices often suffer from inefficiency due to limited output performance and rigidity. To address this issue, a dielectric‐ferroelectric boosted US‐TENG (US‐TENG_DF‐B_) capable of producing a high output charge with low‐intensity ultrasound and a long probe distance is developed, comparatively. The feasibility and output stability of this deformable and augmented device is confirmed under various bending conditions, making it suitable for use in the body's curved positions or with electronic implants. The device achieved an output of ≈26 V and ≈6.7 mW output for remote charging of a rechargeable battery at a 35 mm distance. These results demonstrate the effectiveness of the output‐augmented US‐TENG for deep short‐term wireless charging of implantable electronics with flexing conditions in curved devices such as future total artificial hearts.

## Graphical abstract

Implantable devices rely on batteries that demand surgical replacement, posing risks, and financial burdens. Ultrasound energy transfer (US‐ET) offers a revolutionary wireless alternative but struggles with efficiency. The presented dielectric‐ferroelectric‐boosted US‐TENG (US‐TENG_DF‐B_) is thin, flexible, and biocompatible that provides high‐efficiency and stable power delivery in curved positions, fostering a future of noninvasive and sustainable wireless energy transfer solutions for biomedical applications.

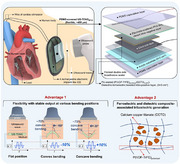

## 1 Introduction

Power discharging in electronic implantable medical devices (IMDs) has been concerning as a medical safety challenge. IMDs are used in wide outcomes of beneficial diagnostic and therapeutic applications of diseases or injuries such as neurostimulation therapy and cardiovascular curing/monitoring.^[^ ^1^, ^2^ ^]^ Additional achy surgeries for battery replacement can lead to patient complications such as surgical site infections, biofilm formation, and high healthcare costs.^[^ ^3^, ^4^ ^]^ These issues have driven substantial research into developing wireless rechargeable and self‐powered/battery‐less IMDs.^[^ ^5^, ^6^ ^]^ Assessments of battery‐less or self‐powered IMDs suggest that converting ambient energies to functional electrical power may be inadequate for all bio‐applications. For example, the mechanical and biochemical energy from the human body might not consistently provide sufficient power for IMDs with complex and high‐energy‐demand treatments.^[^ ^7^ ^]^ In contrast, wireless rechargeable IMDs can provide reliable and manageable energy to support advanced functions,^[^ ^8^ ^]^ though challenges remain in minimizing device/battery size, flexibility, biocompatibility, and feasibility for various implant positions.^[^ ^9^ ^]^ Several innovative energy transfer systems have been developed for wirelessly powering rechargeable IMDs, including electromagnetic induction (radio frequency), electromagnetic irradiation (photovoltaic), and ultrasound energy transfer (piezoelectric, triboelectric).^[^ ^10^ ^]^ Evaluations of wireless energy transfer (WET) strategies suggest that photovoltaic WET is unsuitable due to issues with low light penetration inside tissue and thermal tissue damage, respectively.^[^ ^11^ ^]^ Inductive coupling^[^ ^12^ ^]^ and capacitive coupling^[^ ^13^ ^]^ WETs are appropriate for powering hypodermic electronic implants and data communication devices, respectively. However, they are only suitable for efficient wireless power output or deep energy transmission due to the high‐intensity electromagnetic field required which can cause detrimental tissue.^[^ ^14^ ^]^ Ultrasound waves, as remote mechanical energy sources, can penetrate tissues deeply, making them suitable for a wide range of clinical percutaneous applications in a noninvasive and biocompatible intensity range (by considering FDA guidelines for ultrasound protocols, *I_SPTA_* = 0.72 W cm^−2^) to avoid any thermal or mechanical bioeffects caused by ultrasound energy.^[^ ^15^ ^]^ Ultrasound energy transfer using piezoelectric and triboelectric nanogenerators (US‐PENGs and US‐TENGs) is a well‐recognized technology for converting ultrasound mechanical energy to electrical power for IMDs charging.^[^ ^16^ ^]^ US‐TENGs generally incorporate greater biocompatibility and flexibility due to the variant materials that can be employed. In contrast, US‐PENGs commonly rely on lead‐based piezoelectric ceramics, which are often rigid and may pose toxicity concerns. Many types of US‐PENGs have been developed for powering electronic implants while US‐TENGs generally provide higher power output. A flexible US‐PENG has recently been reported to produce an output of 15.4 µW at a 2.5 cm depth under 0.3 W cm^−2^ ultrasound intensity (in soft tissue).^[^ ^17^ ^]^ However, a non‐flexible ferroelectric‐boosted US‐TENG was presented earlier that could confirm an output of ≈8.5 mW at 3 cm depth under ≈0.2 W ultrasound intensity (underwater).^[^ ^18^ ^]^

In this article, we propose a new advanced US‐TENG designed to extend the temporal functionality of IMDs, especially for required wireless charging applications.^[^ ^19^, ^20^ ^]^ This biocompatible‐covered device with a thickness of 0.4 mm, achieves a high charge density suitable for powering electronic devices up to ≈6 cm deep at noninvasive ultrasound intensities. Indeed, this robust triboelectric technology incorporates a dielectric composite to reduce charge impedance and enhances ferroelectric polarization to improve contact electrification by tuning the work functions of the new match of high‐potential tribo‐layers. The feasibility of the dielectric‐ferroelectric boosted US‐TENG (US‐TENG_DF‐B_) was confirmed at different bending positions and misalignment angles, marking a significant advancement in WET technology. This development is particularly notable for its compatibility with the complex geometries of body organs and the potential for misaligned ultrasound probes with implanted devices. Overall, this bendable device demonstrates performance in charging a battery at different positions compared to the latest WET technologies for IMDs.

## 2 Material and Structural Advantages of US‐TENG_DF‐B_ for Powering Advancement

**Figure 1:**
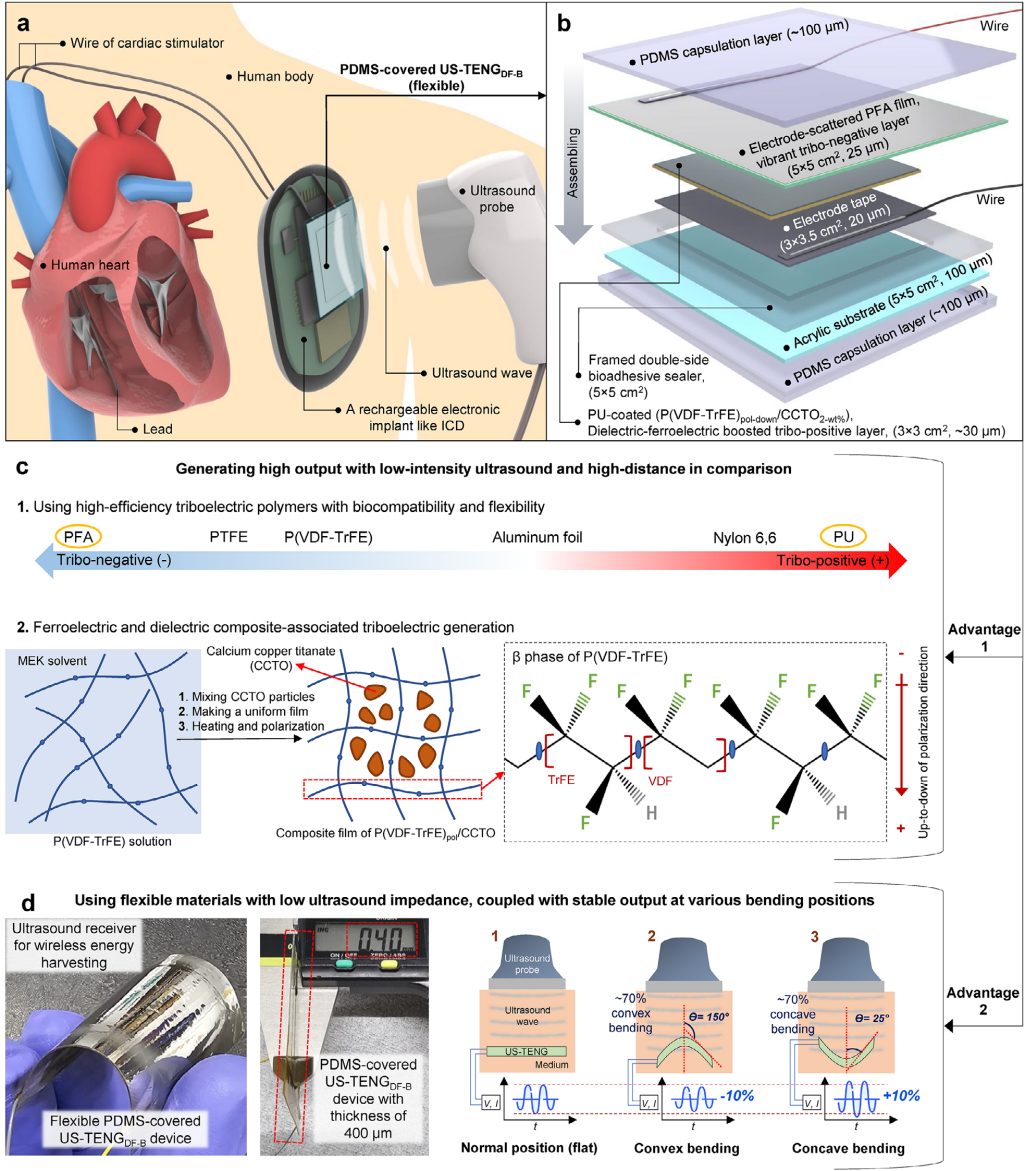
An overview of the proposed US‐TENG_DF‐B_ with material‐structure specifications and advantages. a) Schematically illustration for wireless powering a cardiovascular IMD such as ICD by US‐TENG_DF‐B_. b) Schematic of an exploded detailing structure of flexible US‐TENG_DF‐B_ (5 × 5 cm^2^ overall‐size, 3 × 3 cm^2^ active‐area) with key materials labelled. The dimension and thickness of each layer are characterized individually. The tribo‐positive layer (PU) is booted via composite film of a dielectric (CCTO) and poled ferroelectric (P(VDF‐TrFE)). c and d) The classified main advantages of the US‐TENG_DF‐B_. The initial advantage, utilizing tribo‐layer with high surface charge density and dielectric‐ferroelectric boosted triboelectric. A triboelectric series demonstrates a charge density comparison of used tribo‐negative (PFA) and tribo‐positive (PU) materials with trends (PTFE, Nylone 6,6) that efficiently improve the output performance. A uniform flexible film of dielectric‐ferroelectric composite from well‐mixed P(VDF‐TrFE) solution with CCTO powder, that exposed heating and polarizing to harvest the β‐phase of P(VDF‐TrFE)_pol_/CCTO film. The detailed relevant reports are provided in supplemental information and main text. The second advantage, the photographic images show the flexibility of thin US‐TENG_DF‐B_ (0.4 mm thickness), and a purposeful schematic to classify the implanting positions (flat, convex, and concave) of the device inside tissue with feasible output.

A thin implantable device was designed to generate triboelectric power remotely using ultrasound energy (US‐TENG) for wireless powering of IMDs, such as implantable cardioverter defibrillators. This device integrates modified charge density delivery for interventional electronic implants (**Figure** **1**a). Thin polymeric films were used to create a fully flexible US‐TENG, as shown in Figure 1b. Here, the materials and composites used in the device are introduced, followed by an explanation of the reasons for their selection in subsequent sections. A popular commercial polymer in the biopharmaceutical industry,^[^ ^21^ ^]^ acrylic or poly‐(methyl methacrylate) (PMMA), was selected for the substrate film. The film had an area of 5 cm × 5 cm and a thickness of 100 µm. Perfluoroalkoxy alkanes (PFA), known for their high triboelectric performance under mechanical energy, were used in the design.^[^ ^22^ ^]^ To maintain the unique properties of the PFA film, a nano‐scale layer of electrode was deposited. Au and Ag could be scattered, and identical electric results were yielded through both in the same devices. Au is more suitable option for long‐term device implantation due to its biocompatibility. However, in this study, we primarily scattered Ag electrodes because of availability. The US‐TENG primarily consisted of a mixture of a polarized ferroelectric polymer and dielectric particles, polyvinylidene fluoride‐trifluoroethylene, and calcium copper titanate (P(VDF‐TrFE)_pol_/CCTO) to enhance charge density generation of the triboelectric layer. This composite film was covered with a robust tribopositive layer of polyurethane (PU) to further increase electric charge production. The components were assembled and made electrically conductive using double‐sided adhesive electrode tape to attach the PU‐coated (P(VDF‐TrFE)_pol_/CCTO) layer to the acrylic substrate. A framed double‐sided adhesive tape which is biocompatible in medical applications, was used to seal the device by affixing the electrode‐coated PFA film to the acrylic substrate. The entire device was then encapsulated in a polydimethylsiloxane (PDMS) solution for waterproofing. Elastomeric PDMS (1:20) was used for its biocompatibility, flexibility, and transparency.^[^ ^23^ ^]^ The fabrication process is detailed in Figures S1 and S2 (Supporting Information). This flexible US‐TENG_DF‐B_ was tested for performance in water, polymer/hydrogel, and porcine tissue.

Figure 1c,d illustrates the advantages of the US‐TENG_DF‐B_ in achieving the physical and chemical goals of this study. The strong electrostatic interactions between the tribo‐negative PFA and tribo‐positive PU enable sufficient AC power generation in contact‐separation mode. PFA, with its electron‐withdrawing fluorine groups, and PU, with its electron‐donating methylene diphenyl diisocyanate groups, contribute to effective charge generation.^[^ ^24^ ^]^ PFA film is noted for its mechanical stability and biocompatibility, making it suitable as an ultrasonic vibrant film.^[^ ^25^ ^]^ PU's flexibility and biocompatibility further facilitate integration with IMDs.^[^ ^26^ ^]^ The energy harvesting performance of the US‐TENG is enhanced by an augmentation agent beneath the PU layer a composite film of P(VDF−TrFE)_pol_/CCTO that improves electrification via the ferroelectricity of polarized P(VDF‐TrFE)^[^ ^27^, ^28^, ^29^ ^]^ and reduces charge impedance through the high capacitance of CCTO particles^[^ ^30^, ^31^ ^]^ (Figure 1c). The electrical properties (dielectricity‐ferroelectricity) of P(VDF‐TrFE)/CCTO composite films after polarization have been studied for more understanding behavior of the mixture materials (Figures S3 and S4, Supporting Information). Biocompatibility of the P(VDF‐TrFE)/CCTO composite in terms of cytotoxicity and genotoxicity has been confirmed.^[^ ^32^ ^]^

Despite concerns about the feasibility of the implantable US‐TENG_DF‐B_ in bending/deforming positions and the challenge of thickness minimization, its components were effectively combined to create a flexible and thin device (≈0.4 mm thick) while maintaining semi‐stable electricity harvesting capability, as shown in Figure 1d. Electrical output was evaluated for concave and convex deformations (toward the ultrasound probe) compared to the normal or flat positions. The curvature of the device did not significantly affect output; in fact, the concave position yielded higher output than the flat position due to enhanced trapping of ultrasound waves in the bowl‐shaped device and increased wave collisions on the device's surface. However, output was slightly reduced in convex bending due to mechanical tension in the PFA film and a decrease in vibration ability.

## 3 Mechanism of Dielectric‐Ferroelectric Boosted TENG

**Figure 2:**
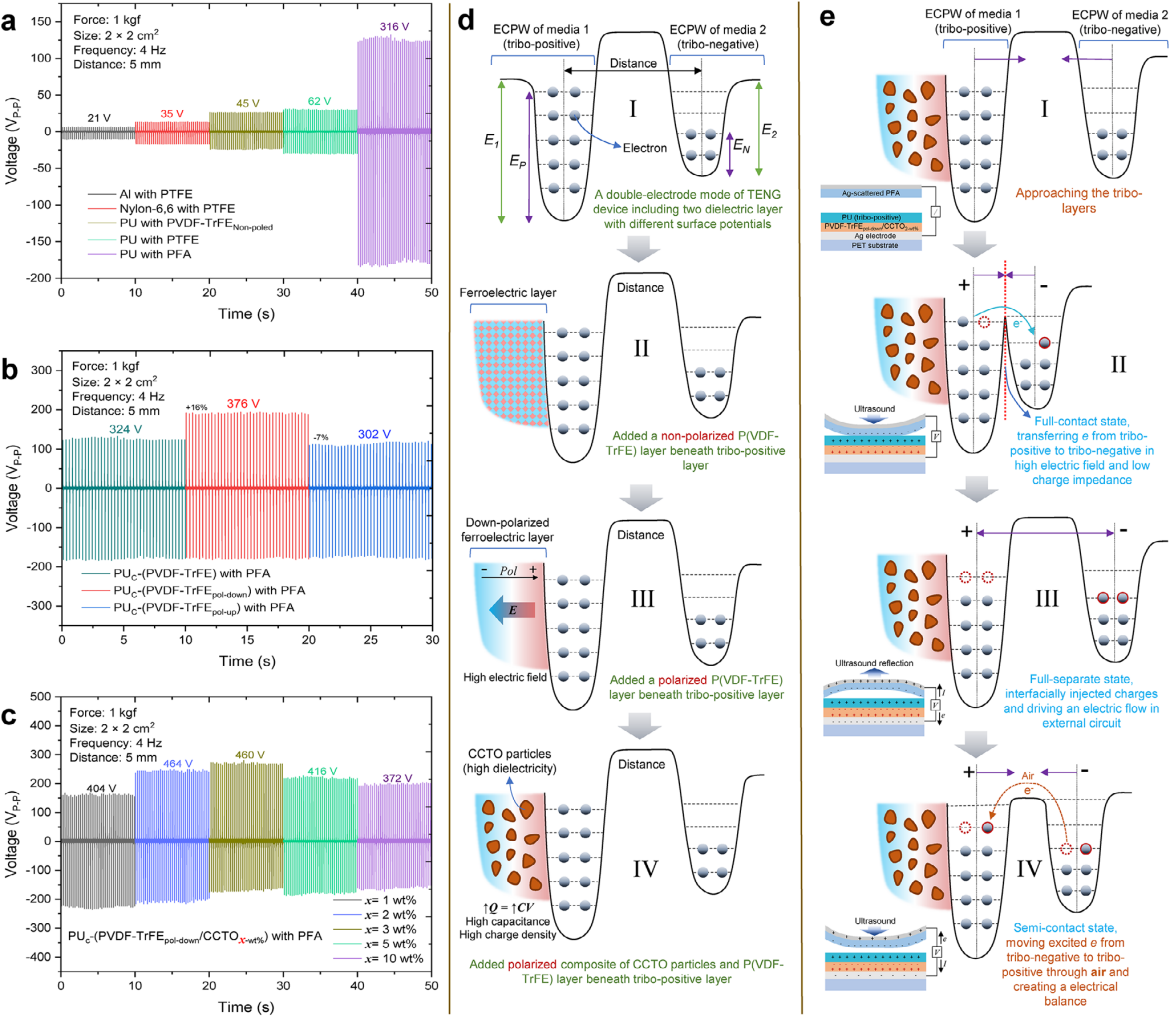
Experimental confirmation and theoretical definition for dielectric‐ferroelectric boosted triboelectric generation. a–c) The results of the voltage output by contact‐separation of the pushing‐test machine, a) comparison of tribo‐layers match that contact‐separation of PU and PFA films could sufficiently harvest higher electrical charge, b) comparison of boosted tribo‐layers of non‐poled, poled‐down, and poled‐up of PU‐coated P(VDF‐TrFE) with PFA that confirmed the downward polarization (poled‐down) direction is adequately improve power harvesting. c) comparison of boosted tribo‐layers of PU‐coated (P(VDF‐TrFE)_pol‐down_ /CCTO_x‐wt.%_), with PFA that demonstrate the evaluated 2 wt.% concentration of CCTO is optimized for output improvement by reducing charge impedance of ferroelectric layer. d) Electric harvesting mechanism in the states of electron‐cloud potential‐well (ECPW) model for contact‐separation mode of tribo‐positive and tribo‐negative materials with different composites of ferroelectric‐dielectric boosted. e) States of ECPW model for an associated TENG with a composite of dielectric and ferroelectric.

To assess the roles of the materials used, an experiment was conducted in the contact‐separation triboelectric mode using a pushing machine to evaluate various tribo‐material candidates and dielectric‐ferroelectric‐boosted triboelectric devices. Initially, well‐known triboelectric material combinations were compared to establish a tribo‐series based on different voltage outputs. As confirmed from the voltage‐pick results presented in **Figure** **2**a, a match of PFA with PU could harvest a significantly large voltage value by mechanical force. This is because of the electrification density on the contact surfaces of tribo‐matches that is generated from strong electron‐negativity of PFA's fluorine and electron‐positivity of PU's methylene diphenyl diisocyanate groups (structurally verified by Fourier‐transform infrared spectroscopy (FT‐IR) in Figure S5a, Supporting Information). Furthermore, the surface potentials of the selected tribo‐layers were measured by Kelvin probe force microscopy (KPFM) to verify comparisons, as results are shown in Figure S6 (Supporting Information). Polytetrafluoroethylene (PTFE) is a popular and efficient tribo‐negative polymer while PFA shows stronger tribo‐negativity performance than PTFE due to its trifluoromethyl groups, which enhances surface energy by charge trapping efficiency. Boosting ferroelectric material with molecular dipoles can improve the electron transport kinetic in the triboelectric generation mechanism. Indeed, attached ferroelectric beneath the tribo‐layer is a modification method for tuning work functions of the tribo‐layer that causes electron mobility through conditional matched polarization direction of the ferroelectric layer with the electric dipole moment of the triboelectric layer.^[^ ^31^ ^]^ Here, beta‐phase P(VDF‐TrFE) or β‐P(VDF‐TrFE) was used as a flexible ferroelectric copolymer matrix with a unique structure, that fluorine is positioned on one side of the carbon‐carbon backbone and hydrogen on the other side^[^ ^33^ ^]^ (Figure S7a,b, Supporting Information). The β‐P(VDF‐TrFE) as a polarized state was obtained by applying an external electric field (Figure S7c, Supporting Information), and its structure was confirmed by FT‐IR and X‐ray diffraction (XRD) analyses as presented in Figures S5b and S8 (Supporting Information). Tree different states of P(VDF‐TrFE) were used to evaluate the performance of ferroelectric‐boosted triboelectric, including upward‐polarized, downward‐polarized, and non‐polarized. The downward‐polarized state of the β‐P(VDF‐TrFE) layer was found to enhance triboelectric power density due to the alignment of molecular dipoles in the positive‐negative direction, as shown in Figure 2b by the voltage results. The differences between upward‐polarized and downward‐polarized ferroelectrics are schematically illustrated in Figure S7c (Supporting Information). In the case of downward polarization, the molecular dipoles in the ferroelectric layer align in the same direction as the electric dipoles of the triboelectric layer, enhancing charge transfer and increasing the electric potential difference across the layers. This alignment creates a strong internal electric field that facilitates efficient charge separation, leading to output performance. In contrast, upward polarization causes dipole alignment in the opposite direction to the triboelectric layer's electric dipoles. This misalignment weakens the internal electric field and reduces charge separation efficiency. As a result, upward‐polarized samples show diminished performance compared to their downward‐polarized counterparts. Unpolarized samples lack a preferred dipole orientation, resulting in a more balanced interaction. While their performance is better than upward‐polarized samples due to the absence of destructive dipole interference.^[^ ^18^, ^34^ ^]^

The charge‐transferring density in friction materials can be enhanced by utilizing high dielectric composites to optimize capacitance and surface potential properties.^[^ ^35^ ^]^ As a high‐dielectric ceramic, CCTO particles were added to the P(VDF‐TrFE) layer to amplify the internal polarization owing to their high permittivity. This strong polarization facilitates more efficient charge separation and movement, crucial for storing electrical charge, which is essential for the device's energy harvesting capability.^[^ ^36^, ^37^ ^]^ Figure 2c illustrates the relationship between voltage output and CCTO concentration. An optimized concentration of ≈2 wt.% significantly improved the voltage output of the TENG. Video S1 (Supporting Information) provides a demonstration of the peak‐to‐peak voltage generation through an oscilloscope screened by contact‐separation of pushing test for comparing the performance of the tribo‐layers that confirms Figure 2a–c data.

The crystal structures of P(VDF−TrFE)_pol_/CCTO composite were characterized by FT‐IR and XRD (Figures S5b and S8, Supporting Information); additionally, micro‐scale amorphic morphology of CCTO particles was observed through scanning electron microscopy (SEM) (Figure S9, Supporting Information). A high concentration rate of CCTO can enhance surface potential, but excessive concentration leads to surface morphology changes in the P(VDF‐TrFE) film that causes full‐contact deficiency by protrusions on the friction surface and resulting output dropping; the KPFM images with different values of surface potential and roughness for confirming impression of with/without CCTO boosted in polarized/non‐polarized P(VDF‐TrFE), are shown in Figures S10 and S11 (Supporting Information).

In triboelectric devices, surface friction plays a dual role, influencing both charge generation and contact efficiency. Moderate friction is essential for effective charge transfer because it enhances contact electrification during the triboelectric process. However, excessive surface friction often caused by surface protrusions or irregularities can disrupt uniform contact between triboelectric layers. This disruption creates localized areas with reduced or no contact, leading to a drop in charge transfer efficiency. In the case of US‐TENG_DF‐B_, we observed that engineered surface features optimize charge transfer while minimizing negative impacts. The P(VDF‐TrFE)/CCTO layer was designed to maintain smoothness at the microscopic level (≈1 µm) while providing high surface energy for electrification. When surface protrusions were experimentally introduced beyond a certain concentration of CCTO powder (after 2‐wt.% up to 5‐wt.%) while the surface potential was almost constant, it negatively affected performance by causing contact inconsistencies.

To understand the strategy of incorporating dielectric and ferroelectric in triboelectric technology for enhancing electric charge generation, the states of the electron‐cloud potential‐well (ECPW) model schematically presented that begins for a contact‐separation mode of typical tribo‐positive and tribo‐negative materials with different work functions (Figure 2dI). The next steps are showing a comparison of boosted ferroelectric to tribo‐positive after and before polarization of ferroelectric by an external electric field (Figure 2dII,III), where the manipulated work function of the highest occupied molecular orbital (HOMO) level of the polarized ferroelectric facilitates electron transfer through a high internal electric field. Using a high‐dielectric material effectively reduces the impedance value and enhances the surface charge density due to high capacitance (Figure 2dIV). Additionally, the steps in the energy harvesting mechanism of associated TENG with a composite of dielectric and ferroelectric are conceptually illustrated through the ECPW model in Figure 2e. First, a TENG_DF‐B_ device is exposed to the mechanical force (Figure 2eI); and the friction tribo‐layers move closer to each other until a full‐contact state is reached as a result of electron transfer from the tribo‐positive to the tribo‐negative because of the chemical potential or Fermi energy level (Figure 2e(II)). Therefore, the electron vacancies are occupied through interfacially injected electrons and driving an electric charge flow in an external circuit until a fully separate state is reached (Figure 2eIII). Subsequently, the excited electrons move from tribo‐negative to tribo‐positive through the air to make a partial balance of electric charge in a semi‐contact state (Figure 2eIV). These steps are repeated respectively based on the frequency of mechanical contact‐separation motion.^[^ ^38^, ^39^ ^]^

## 4 Conditional Ultrasound‐Responsive Output Performance

**Figure 3:**
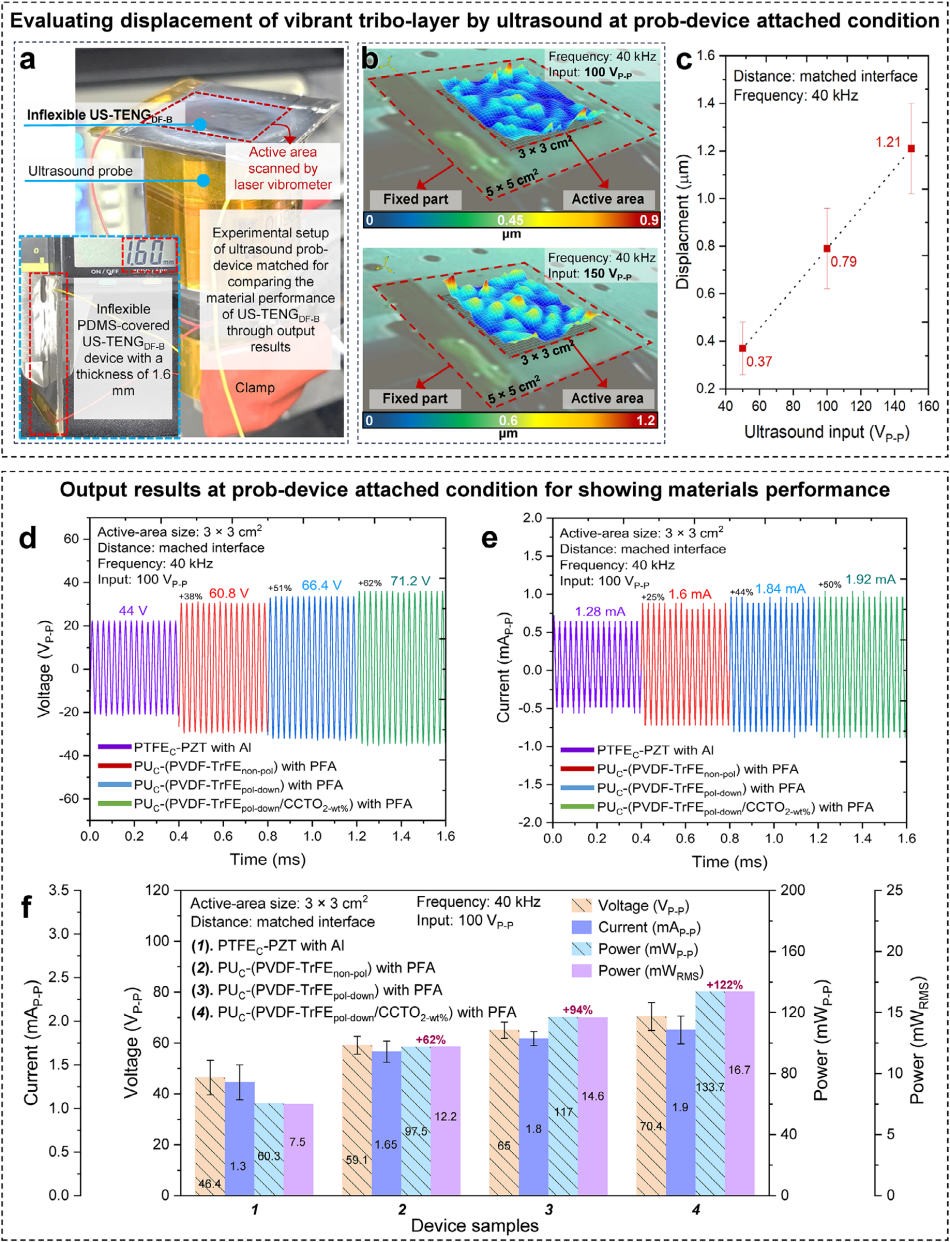
Experimental setup and ultrasound‐driven output results at prob‐device matched condition. a) Photograph of the setup under constant experimental parameters of ultrasound/device to value selected composite. b) The surface displacement scan images of the vibrant PFA film by ultrasound wave that was measured through a laser vibrometer at sinusoidal wave condition of 100 V_P‐P_ and 150 V_P‐P_ inputs. c) Displacement measurement at different ultrasound inputs/intensities. d–f) Overall composite performance evaluation results of US‐TENG_DF‐B_ at prob‐device matched condition. d) Synodical voltage pikes, e) synodical current pikes, f) voltage‐current‐power‐power_RMS_ outputs comparison of devices including tribo‐layers matches that ultrasound‐driven contact‐separation of PU‐coated (P(VDF‐TrFE)_pol‐down_ /CCTO_2‐wt.%_) and PFA film is sufficiently charge generation augmented.

**Figure 4:**
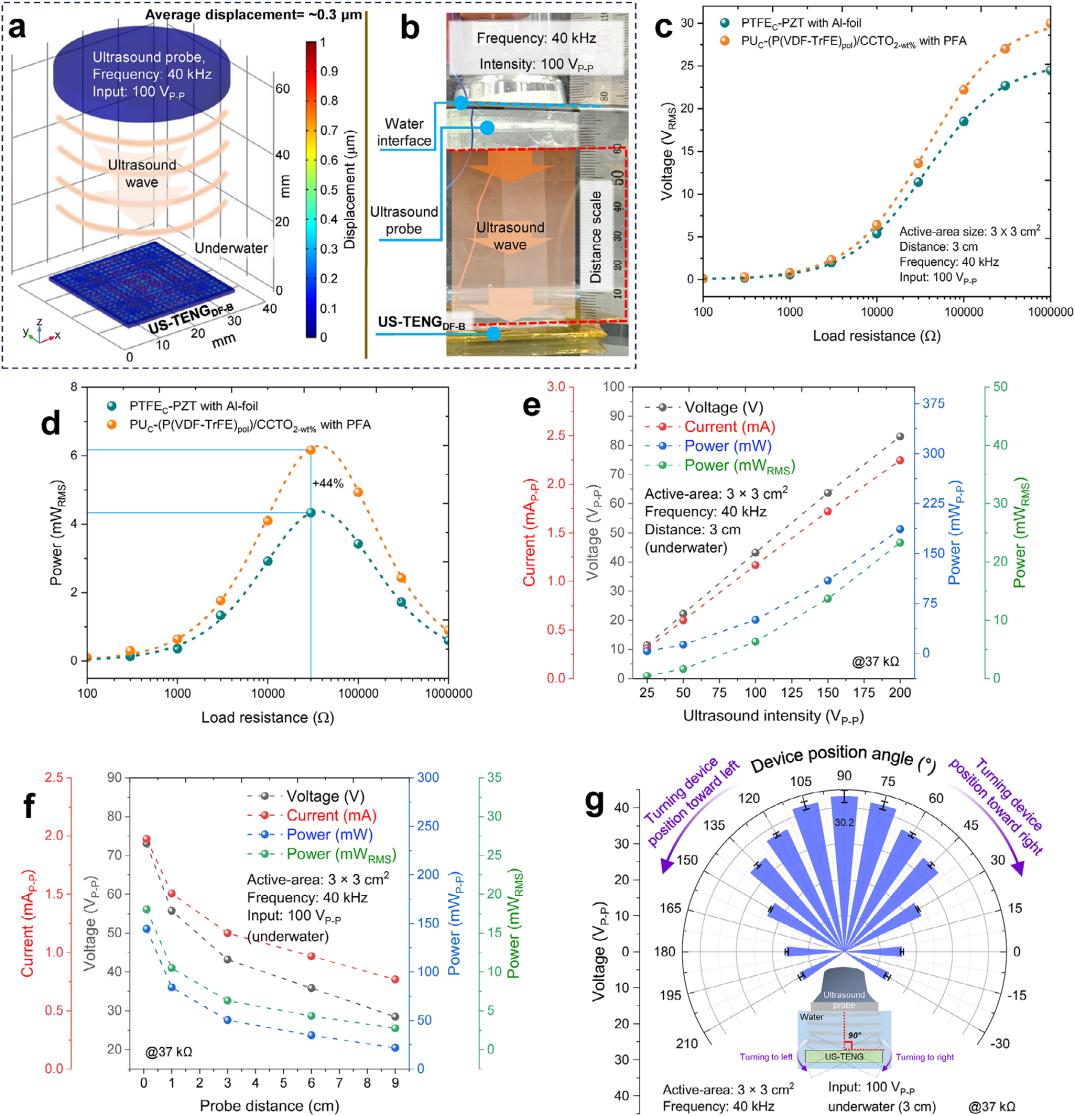
Simulation, experimental setup, and ultrasound‐driven output result in underwater conditions. a) A simulation result to demonstrate feasible displacement and vibration of PFA film under ultrasound energy. b) Photograph of the setup under different experimental parameters of ultrasound/device to optimize device performance. c–g) Overall experimental parameters evaluation results of US‐TENG_DF‐B_ at underwater condition. c) The graph of tuned voltage_RMS_ output and d) the graph of tuned power_RMS_ output at different load resistances for obtaining resistance optimization of the highest output point and comparing US‐TENG_DF‐B_ with the previously reported device. The RMS value of power was driven through $\frac{V_{RMS}^{2}}{R_{\left(\Omega \right)}}$. The graph of voltage‐current‐power‐power_RMS_ output with 37 kΩ at e) different ultrasound intensities (25 – 200 V_P‐P_ inputs) and f) different probe distances (0.1 – 9 cm). g) The graph of voltage output at different device‐probe angles (0–120 degrees of left‐right turning). The water container was grounded during measurements to isolate the observed output from any spurious electrical conduction effects.

The electrical output results from the US‐TENG_DF‐B_ device are demonstrated in **Figure** **3** which proceeded at conditions of probe‐device attached and underwater for checking conditional power harvesting feasibility based on constituent materials under tuned physical parameters during the experiments. Figure 3a presents the experimental setup, where the ultrasound probe was interfaced with the inflexible, rigid side of the device to achieve ultrasound‐responsive feedback. For this setup, the device's substrate was thickened to 1.2 mm, ensuring it remained completely flat on the active ultrasound probe, which aided in measuring the resonant displacement of the electrode‐scattered PFA layer using a laser vibrometer. The efficiency of vibrational displacement plays a key role in enhancing power harvesting by promoting the mechanical contact separation of friction layers. The displacement value was linearly amplified with increasing ultrasound intensity or voltage input, as illustrated in Figure 3b,c. The study of ultrasound‐driven outputs was conducted using consistent experimental factors: a 3 cm × 3 cm active device area, an operating frequency of 40 kHz, and an ultrasound input of 100 V_P‐P_. Figure 3d,e displays the voltage and current signals of devices composed of different materials. A comprehensive stepwise comparison of voltage, current, power, and power_RMS_ outputs confirmed over 120% improvement in power development, showcasing the performance of the US‐TENG_DF‐B_. This device utilized a composite of PU‐coated (P(VDF‐TrFE)_pol‐down_ /CCTO_2‐wt.%_) with PFA (Figure 3f). The 120% improvement was calculated by comparing the peak power outputs of the US‐TENG_DF‐B_ with previously reported triboelectric nanogenerators under equivalent experimental conditions. For the benchmark of our device, the peak power output of the reference device was 7.5 mW_RMS_, using a similar ultrasound intensity and probe distance. In comparison, the US‐TENG_DF‐B_ demonstrated a peak power output of 16.7 mW_RMS_ under the same conditions. The percentage improvement is calculated as follows: improvement (%) = [(Power_US‐TENGDF‐B_ – Power_Reference_) / Power_Reference_] × 100. Underwater experimental conditions physiologically enable ultrasound biomedical research by relying on the close sound impedance values of water and soft human body tissues, which is a further model evaluation approach to assessing ultrasound‐responsive output performance.^[^ ^40^ ^]^ However, encapsulating electronic devices to overcome waterproofing challenges in underwater environments must be considered.^[^ ^41^ ^]^ In this study, an ultrathin PDMS layer, considered a reliable encapsulation material,^[^ ^42^ ^]^ was used to cover the entire surface of the US‐TENG_DF‐B_ to make it a waterproof device. The results of a simulation demonstrated the feasible mechanical displacement of the vibrant PFA film under ultrasound energy, which is visible through an up‐down gradient on a color scale (**Figure** **4**a). An image of the underwater experimental setup is shown in Figure 4b. The position of the ultrasound receiver device could be easily changed in water to further explore the feasibility of the device at different distances and angles. The voltage and power in RMS value with adjusted load resistance measurements presented in Figure 4c,d to confirm ≈44% in enhanced gain of the US‐TENG_DF‐B_ compared to that reported last version of the similar category technology.^[^ ^18^ ^]^ Furthermore, conditional voltage, current, power, and power_RMS_ output at 37 kΩ were measured at different ultrasound intensities (25 – 200 V_P‐P_ inputs), different probe distances (0.1 – 9 cm), and voltage at different device‐probe angles (0 – 120 degrees) (Figure 4e–g) to assess the dependence of the device output efficiency on relevant environmental and physical factors.

## 5 Feasible Confirmation in Bending Positions

**Figure 5:**
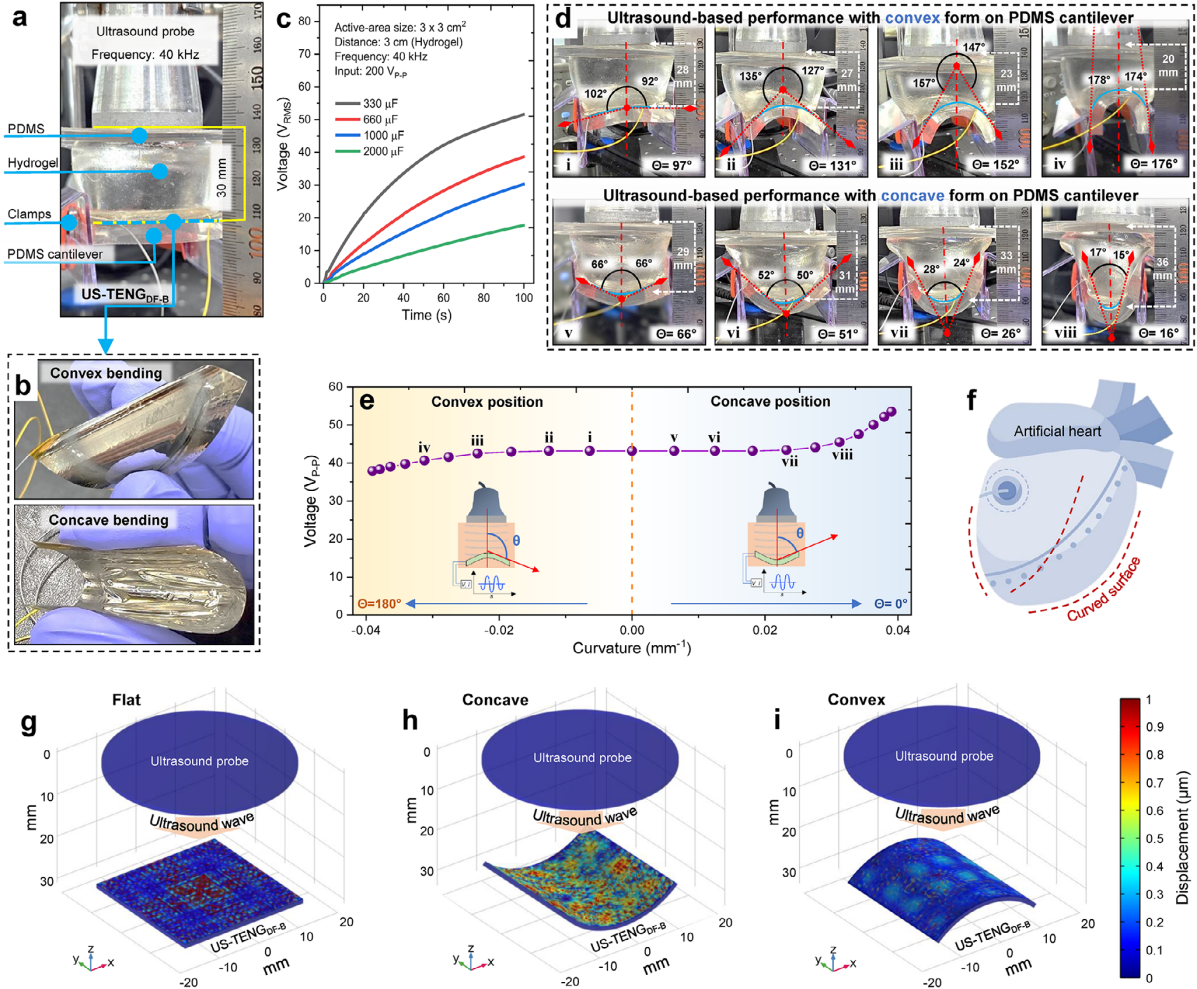
Feasible confirmation of ultrasound‐driven electric harvesting with mechanical bending conditions under soft media. a) Overall view of the experimental setup for exerting mechanical deformations by clamping the flexible supporter‐device attached under hydrogel/PDMS‐probe matched. b) Real images of US‐TENG_DF‐B_ at manual convex and concave deformations. c) Open‐circuit output voltages for charging different capacitors (330 – 2000 µF) at 40 kHz and 200 V_P‐P_ of ultrasound, 3 cm distance, and flat (usual) position. d) The optical images of tuned bending degrees of the installed device with visually measured angles and probe‐device distances. e) Peak‐to‐peak voltage results in terms of mechanical convex and concave deformations of the device. The parameter of hypothetical circle radius (r) on flexed plate computationally was extracted from the central‐fixed angle of two curved edges that theatrically is explained in Figure S12 (Supporting Information). f) A schematic example of a power‐demand artificial heart as a future curved electronic implant. g–i) The simulation results of the generated ultrasound pressure field onto the flat, convex, and concave positions of the US‐TENG_DF‐B_ at 3 cm probe‐device distance, for comparing their displacement gradient of vibrant PFA film.

To demonstrate the practicality of the US‐TENG_DF‐B_ device under flexed conditions, voltage outputs were recorded while mechanically deforming the device to various degrees of curvature. A system was developed to simulate ultrasound induction on the flat PDMS‐attached US‐TENG_DF‐B,_ using a hydrogel∖PDMS medium. Relying on their flexibility, the device can become concave and convex shapes with several bending degrees through horizontal adjustment of clamps (**Figure** **5**a,b). In this setup, hydrogel and PDMS were selected for their stretchable and flexible properties, mimicking the ultrasonic behavior of human soft tissues. Hydrogel served as an adhesive layer to uniformly cover the device surface, while non‐adhesive PDMS, embedded between the probe and hydrogel, acted as a surrogate for skin.^[^ ^43^ ^]^ PDMS also functioned as a bottom support, preventing a wavy shape during curvature adjustments. In the initial measurements, power harvesting was tested in the flat position by charging different capacitors in an open circuit configuration. The resulting voltage‐time outputs are presented in Figure 5c. To further validate the device's performance in curved states, images of the setup were recorded, and mathematical tools were employed to approximate the degree of curvature by measuring the central fixed angle between the device's edges (Figure 5d). The curvature was manually adjusted to become either concave or convex, directed toward the ultrasound probe. The hypothetical circular radius (r) for the curved device was calculated from the angle values to define the deformation parameters comprehensively (Figure S12, Supporting Information). Voltage output for each curvature state was recorded, with the results displayed in Figure 5e. The device showed minimal changes in output for up to ≈70% convex‐concave bending (−0.65 to 0.65 of r⁻¹, corresponding to ≈25–150° of curvature) compared to the flat position. This flexibility in output performance is crucial for potential applications in curved electronic implants, such as a total artificial heart, as schematically indicated in Figure 5f. At extreme bending, slight output variations were observed: convex formations with ≈70% bending caused an output drop of ≈10%, while concave formations under similar conditions enhanced the output by up to ≈10%, as seen in Figure 5e. Here, the consumed polymers with low ultrasound impedance mismatch with biological tissues ensure efficient ultrasound energy transfer, while their flexibility maintains reliable performance in mechanical deformation and prevents output fluctuations. The measurements were set at 3 cm probe‐device distance (flat poison), frequency of 40 kHz, and an ultrasound input of 0.2 W cm^−2^. Furthermore, simulations of US‐TENG_DF‐B_ specimens in flat, convex, and concave positions were performed, assuming the same ultrasound pressure field, material characteristics, active area (3 × 3 cm^−2^), and probe distance (3 cm), for comparing displacement of vibrant PFA film (Figure 5g–i). The results confirm greater displacement in the concave state than in other positions under the same conditions, owing to ultrasound energy being trapped in the bowl‐shaped curved device, which causes greater vibration. The lower displacement in the convex state is due to mechanical tension in the vibrant PFA film, which slightly reduces vibration.

## 6 Ex Vivo Tests for Showing Wireless Powering and Stable Output at Different Curvatures

**Figure 6:**
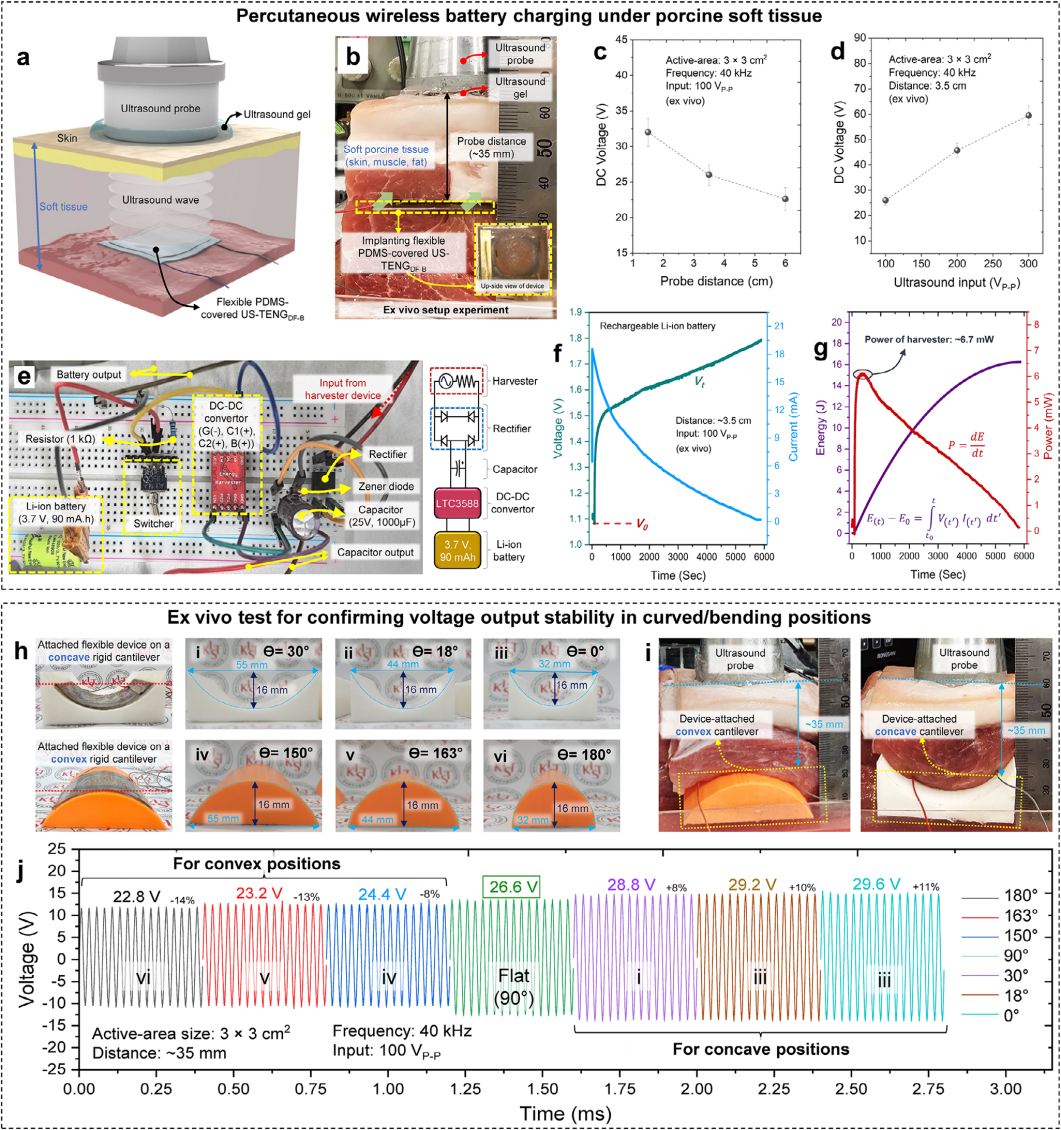
Setup of ex vivo test and curved device performance results under porcine tissue. a) A schematic illustration and b) a real photo of the ex vivo experiment for evaluating the feasibility of the ultrasound‐driven implanted device under soft tissue. The voltage output (directly measured) c) at different ultrasound intensities, and d) at different probe distances. e) A assembled circuit board for battery powering. f) The real‐time rated voltage‐current output curve of the battery charging, and g) the calculated energy‐power curve of the charging; arranged data of supplied voltage‐current on the battery (charger's dynamic response). h and i) The photos of the device‐attached concave and convex rigid cantilevers with different curvature degrees and relevant ex vivo setup. j) Diagram of the voltage amplitudes of ultrasound‐driven device embedded under porcine tissue (≈35 mm, 100 V_P‐P_, 40 k Hz) for different curvature grades of device‐attached cantilevers.

An ex vivo experiment was conducted using soft porcine tissue to confirm wireless battery charging by the implanted US‐TENG_DF‐B_ device_._ Porcine tissue closely resembles human tissue, offering a reliable model for evaluating deep tissue battery charging. Animal models such as rats or rabbits were unsuitable due to insufficient tissue thickness and potential device bending or deformation during implantation. The experimental setup is illustrated in **Figure** **6**a,b with additional details in Figure S13 (Supporting Information). The performance of the device was evaluated by adjusting the intensity and distance of the ultrasound induction (Figure 6c,d; Figure S14, Supporting Information). The alternating voltage signal generated by the US‐TENG_DF‐B_ was converted into direct voltage using a rectifier integrated into the battery's circuit. Figure 6e shows a detailed layout of the circuit components. The resulting rated voltage of battery charging, representing the charger's dynamic response, demonstrates the remote power transfer capability of the device. A Li‐ion rechargeable battery (3.7 V, 90 mA.h capacity) was charged (not fully) with 16 J energy stored for 5800 s at a 3.5 cm distance and a 100 V_P‐P_ (≈0.2 W cm^−2^) ultrasound input (Figure 6f,g).^[^ ^44^ ^]^ The energy storage and power values were derived from the measured voltage‐current curve using $E_{\left(t\right)}-\;E_{0}=\int_{t_{0}}^{t}V_{\left(t^{\prime }\right)}\;I_{\left(t^{\prime }\right)}\;\;dt^{\prime }$ and $P=\frac{dE}{dt}$ respectively. However, we should consider some factors during the charging process. In Figure 6f, the battery voltage exhibits a gradual increase, which aligns with the expected behavior as the battery accumulates charge. However, the current experiences a sharp initial drop instead of maintaining a stable constant current phase. This rapid decline in current can be attributed to multiple factors, including the internal resistance of the battery, the characteristics of the charging circuit (resistance, impedance mismatch, and conversion inefficiencies), or limitations in the power supply. These factors collectively influence the charging profile, resulting in a non‐ideal charging process.

This device exhibited exceptional wireless energy transfer stability, highlighting its potential for reliable consistent power delivery and confirmed for ex vivo and underwater conditions, as shown in Video S2 (Supporting Information). To further assess the device's battery powering efficiency under curved conditions through voltage measurement, rigid convex and concave cantilevers were fabricated using 3D printing (Figure 6h). These cantilevers were used to deform the attached device, simulating real‐world applications under soft tissue conditions (Figure 6i). The results indicated that the concave shape generated more output than the flat position, due to increased displacement caused by trapped ultrasound waves in the bowl‐shaped structure. Conversely, in the convex position, tension in the vibrant PFA film reduced vibration, leading to a decrease in displacement. However, the differences in output between curvature states were not significant and that shows a reliable and stable output, as seen in Figures 6j and S15 (Supporting Information). The charge densities are achieved across varying conditions, at different probe distances and ultrasound intensities for underwater. Also, the charge density generated was evaluated at variant curvatures for underwater and ex vivo conditions that demonstrate the device's ability to consistently generate high charge density under bending positions for efficient wireless energy transferring in deep tissue implant applications. These results highlight the efficiency and robustness of the device for wireless energy transfer in deep tissue implant applications. (Figure S16, Supporting Information).

All measurements adhered to FDA guidelines for ultrasound protocols, with the ultrasound pressure set ≈0.2 W cm^−2^ well below the intensity limits (*I_SPTA_* = 0.72 W cm^−2^) to avoid any thermal or mechanical bioeffects caused by ultrasound energy.^[^ ^15^ ^]^

The US‐TENG_DF‐B_ is a thin and flexible electronic device and it is important to be strong enough under tension that occurs during bending or mechanical deformations. Therefore, Figure S17 (Supporting Information) shows a durable functional property by stable voltage output over multiple convex‐concave bending cycle tests. Additionally, the biocompatibility of PDMS‐capsulated US‐TENG_DF‐B_ was confirmed through in vitro 3‐(4, 5‐dimethylthiazolyl‐2)‐2, 5‐diphenyltetrazolium bromide (MTT) assay tests. The MTT results show high cell viability even after 1, 2, and 3 days (Figure S18, Supporting Information).

## 7 Discussion

An enormous number of battery‐required IMDs have been developed in four functional categories containing neurostimulators,^[^ ^45^ ^]^ cardiovascular interventionists,^[^ ^46^, ^47^, ^48^ ^]^ bone‐growth stimulators,^[^ ^49^ ^]^ and programable drug delivers;^[^ ^50^, ^51^ ^]^ which work with many smart IMDs technologies for curing or monitoring health issues.^[^ ^52^, ^53^ ^]^ (Table S1, Supporting Information). A safe and durable electrical source for wireless battery powering of IMDs is needed that could be beneficially appropriate by solving the implant challenges of minimization, flexibility, and power generation sufficiency.^[^ ^54^, ^55^ ^]^ Such an electrical source could also help prevent patient complications such as surgical site infection and expensive healthcare due to additional battery replacement surgeries.^[^ ^56^, ^57^, ^58^ ^]^ In some cases, long‐term devices such as the total artificial heart require high power to dominate the mechanical energy of the blood‐cycle pump process without outside‐inside body connections.^[^ ^59^, ^60^, ^61^ ^]^ In this study, we leveraged the advantages of ultrasound energy transfer based on triboelectric technology at an important development stage in wireless powering electronic implants. Technically relevant measurements are rigorously prioritized with evidential details. Evaluation in studies about US‐TENG_DF‐B_ (0.4 mm thickness, 3 × 3 cm^2^ active area) which is associated with dielectric and ferroelectric materials for charge‐impedance reduction and electrification enhancement respectively, show a high charge density at 40 kHz and 0.2 W cm^−2^ compared to last updated same‐categorized devices (up to 44% for underwater) in addition to high fidelity in the wireless energy transferring feasibility under deforming/curving positions (up to 60% bending), misalignment angles, and deeper implantations (up to ≈6 cm). Furthermore, a rechargeable battery was charged (not fully) with 16 J energy stored for 5800 s at a 35 mm distance, 40 kHz frequency, and 100 V_P‐P_ ultrasound input. The choice of ultrasound probe distance reflects realistic clinical scenarios, balancing noninvasive functionality and effective energy transfer. Additionally, the 0.4 mm thickness was selected to optimize flexibility, low weight, mechanical bending stability, and mechanical vibration under ultrasound. In conclusion, the current biocompatible device has been augmented with a durable output approach for percutaneous charging at various curvatures of physiological conditions and the reported results confirm that this new thin and flexible US‐TENG_DF‐B_ can potentially be integrated with IMDs which require a power supply of energy to overcome the electrical energy of complex functions in the body.

## 8 Experimental Section

### Materials

Poly(vinylidene fluoride‐co‐trifluoroethylene) copolymer (P(VDF‐TrFE)) powder was purchased from Arkema‐Piezotech FC20. Polyurethane (PU) granules were obtained from GoodFellow. Polydimethylsiloxane (PDMS) with curing agent (3‐aminopropyl)triethoxysilane (APTES) was purchased from Dow Chemical Company. The films of perfluoroalkoxy alkane (PFA), polyamide 6 (Nylon 6,6), polytetrafluoroethylene (PTFE), and acrylic poly‐(methyl methacrylate) (PMMA) were purchased from Hanarotr. Methyl ethyl ketone (MEK) solvent (for P(VDF‐TrFE)) and N,N‐dimethylformamide (DMF) solvent (for PU) were obtained from Sigma‐Aldrich. The constituent materials of consumed hydrogel including acrylamide powder, lithium chloride, N,N′‐Methylenebisacrylamide (MBAA), potassium chloride, ammonium persulfate, and N, N,N″,N″‐Tetramethyl ethylenediamine (TEMED) were obtained from Sigma‐Aldrich.

Calcium copper titanate (CaCu_3_Ti_4_O_12_ (CCTO)) particles were synthesized through a powder processing strategy. Initially, according to the ratio of substances in the CCTO crystal structure, the pure metal oxide powders of titanium tetrachloride (TiCl_4_), calcium carbonate (CaCO_3_), and copper oxide (CuO) as raw materials were well‐mixed to prepare the ceramic powder. The powders were calcined at 900 °C and sintered at 1075 °C for 72 h. The ceramic pellets were milled for 24 h to create the microsized CCTO particles. All raw materials were obtained from Sigma‐Aldrich.

### Fabrication of PU‐Coated P(VDF−TrFE)_pol_/CCTO Film

Initially, P(VDF−TrFE) powder was added to the MEK solvent in a ratio of 20% (w/v) and heated on a hotplate at 100 °C with stirring for 3 h. A homogenous solution was obtained, after which specific concentrations of synthesized CCTO powder (0 – 20 wt.%, P(VDF−TrFE)/CCTO) were added to several glass vials. The mixtures were subjected to sonication at 40% power for 10 min (3 s on, 3 s off) to ensure thorough mixing. The mixtures were then reheated and stirred on the hotplate for an additional 30 min to achieve a well‐homogenized solution. Next, a uniform film was fabricated using an automatic tape‐casting coater equipped with a heater and dryer. The viscous composite solution was poured into a micrometer‐adjustable film applicator, which was fixed onto a rolling polyethylene terephthalate (PET) substrate. Vacuuming was applied to prevent pore formation, and the film was dried at 60 °C to remove the MEK solvent. The film underwent annealing at 150 °C for 1 h, followed by polarization under an external electric field of 14 kV for 3 h. The resulting flexible, 20 µm thick, polarized P(VDF−TrFE)/CCTO film was then physically covered with a sticky PU layer. To create a PU film ≈5 µm thick, PU granules were dissolved in DMF solvent at a concentration of 7% (w/v). The solution was stirred and heated at 90 °C for 2 h. The PU solution was then spin‐coated onto a glass plate at 1000 rpm for 10 s and dried at room temperature for 24 h. The film fabrication process is depicted in Figure S1 (Supporting Information).

### Assembly of PDMS‐Capsulated US‐TENG_DF‐B_

Two versions of the device were fabricated including flexible acrylic (0.1 mm thickness, cut by guillotine cutter) and inflexible acrylic (1.2 mm thickness, cut by ultrasonic cutter) substrates in dimensions of 5 × 5 cm^2^. Then, a double‐sided Ag‐electrode tape (20 µm, 3 × 3.5 cm^2^) was centrally attached to the substrate, and fabricated PU‐coated P(VDF−TrFE)_pol_/CCTO film (25 µm, 3 × 3 cm^2^) stickled to the electrode tape; also, a micro‐wire was connected to extra 0.5 cm area of electrode tap. The same as the dimensions of the substrate, a biocompatible double‐sided adhesive tape (MED6501SI, Avery Dennison) was framed by a cutter with 1 cm edges and a 3 × 3 cm^2^ central removed area which was attached to the substrate for device sealing. Next, PFA film (25 µm, 5 × 5 cm^2^) was electrode deposed in nano‐scale by plasma coater machine for 4 min and it covered the upside of the device by sticking to the framed double‐sided bioadhesive tape. Finally, after connecting the second micro‐wire to the electrode‐scattered PFA film, the whole device was immersed in the PDMS solution (20:1) rinsed off, and dried in the oven at 60 °C for 3 h.

### Electric Properties of P(VDF−TrFE)_pol_/CCTO Composite

First, several devices including the sample with a simple structure were fabricated for analyzing the electric properties of polarized/nonpolarized P(VDF−TrFE)/CCTO composite. A glass substrate (1 mm, 2 × 2 cm^2^) was covered by an aluminum electrode (as a down electrode), and the solution of the sample was spin‐coated on the electrode at 1000 rpm for 10 s and dried at room temperature for 24 h. Then, the micro‐scale silver electrodes (as top electrodes) were deposed to the sample layer by plasma sputtering coater and a mask with square holes the size of each hole was 2 × 2 mm^2^. In the end, the top and bottom needles of the impedance analyzer were fixed on the electrodes of the device. Frequency‐dependent capacitance constant (*ε_r_*) and loss tangent (dissipation factor) (tan*δ*) were measured using an Agilent 4294A LCR meter with a 1 kHz frequency at 0.5 V oscillation level, probe‐station (MS TECH), and a parallel equivalent circuit. The dielectric constant was calculated from the obtained capacitance through *ε_r_* *=* *C d /ε_0_ A*; where *C* is the capacitance (Farads), *d* is the thickness (m) of the samples, *ε*_0_ is the permittivity of free space (8.854 × 10^−12^ F m^−1^), and *A* is the surface area of the capacitor's electrode (m^2^). In addition, the loss tangent was subsequently calculated through tan*δ *= 1 / (2*π f R_p_ C_p_*), where *f* is the frequency, *R_p_* is the equivalent parallel resistance, and *C_p_* is the equivalent parallel capacitance.^[^ ^62^ ^]^ Also, frequency‐dependent polarization–electrical field (P–E) hysteresis was analyzed to study the ferroelectric property of the samples at room temperature by a ferroelectric test system (Precision 4 kV HVI and Precision Premier II (RADIANT); Vision software).

### Characterization of the Materials

The chemical structures of the fabricated films, including polarized P(VDF‐TrFE), polarized P(VDF‐TrFE)/CCTO composite, PU, and the commercial PFA film, were analyzed using a Fourier transform infrared spectrometer (ThermoFisher Nicolet iS10, ATR(Ge), DTGS). The crystal structures of both polarized and nonpolarized P(VDF‐TrFE) films, as well as CCTO powder and their composite states, were identified using X‐ray diffraction (XRD) analysis performed with a Bruker D2 Phaser diffractometer. The particle morphology of CCTO powder was characterized using scanning electron microscopy (SEM, JOEL JSM‐IT100), which provided detailed images of the powder's surface features. Additionally, the surface morphology and surface potential of both polarized/nonpolarized P(VDF‐TrFE) composite films were studied using atomic force microscopy (AFM) (Park Systems, XE‐100, Multi75E‐G tip). For the Kelvin probe force microscopy (KPFM) measurements, a 3 × 3 µm^2^ area was scanned, and data were collected with the AFM tip biased at 2‐V AC amplitude, using a highly ordered pyrolytic graphite (HOPG) sample as a reference.

### Electrical Output Performance

The voltage output signals were measured and screened using a four‐channel digital oscilloscope (Tektronix, DPO4014B Digital Phosphor) with a passive voltage probe (KEYSIGHT‐N2140A, 10:1//200 MHz//10 MΩ). A pushing machine with a linear step motor (VEXTA, PK268‐03B) was employed to apply and release periodic compressive external pressures to compare the outputs based on the advantages of the selected materials. The NMC‐102NLC program was used to control the movement distance (5 mm), frequency (4 Hz), and mechanical force (10 N, 1 kgf).

To harvest electric output from ultrasound energy, a system was established comprising an ultrasonic transducer (FUJICERA‐FBL40452HS), an arbitrary waveform function generator (Tektronix AFG1022) to adjust ultrasound frequency and intensity, and a high‐speed power amplifier (NF‐4010) to boost the signal. To reduce noise interference, the ultrasonic transducer probe was grounded. Both voltage and current outputs were measured, with current signals captured using a low‐noise current preamplifier (SRS‐SR570).

To assess real‐time displacement and acceleration of the vibrant tribofilm under probe‐device‐matched conditions, a digital laser vibrometer (Polytec, PSV‐400) was employed. The scanning head of the vibrometer was positioned ≈40 cm from the device surface, with the laser automatically focused on the center of the device's vibrant area (3 × 3 cm^2^). The active scan area and 200 scan points were controlled and adjusted using Polytec's controller software for precise measurements.

### Modulation of Bending Response of US‐TENG_DF‐B_

The mathematical relationship (*l *=* r·θ*) was applied, where *l* is the arc length, *r* is the radius, and *θ* is the angle of bending, to correct the geometric measurements. Figure S12 (Supporting Information) illustrates the details of this approach. Since the radius of the hypothetical circle aligns with the curved surface of the device, using *r* instead of *θ* provides a more accurate measurement of the bending rate. By applying the Taylor expansion of trigonometric functions Equations ((1), (2), (3), (4), (5), (6)), we were able to determine the radius of curvature *r* and the curvature kappa (κ) (Figure S12a, Supporting Information), enhancing the precision of the analysis.

(1) $$\sqrt{r^{2}-{\left(r-h\right)}^{2}}=r\cdot \sin\left(\frac{\theta }{2}\right)$$

(2) $$r^{2}-{\left(r-h\right)}^{2}=2rh-h^{2}=r^{2}\cdot \sin^{2}\left(\frac{\theta }{2}\right)$$

(3) $$\sin^{2}\left(\frac{\theta }{2}\right)=\frac{1-\cos\left(\theta \right)}{2}$$

(4) $$\cos\left(\theta \right)≅1-\frac{\theta^{2}}{2}$$

(5) $$r\left(h\right)=\frac{h}{2}+\frac{l^{2}}{8\cdot h}$$

(6) $$\kappa \left(h\right)=\frac{1}{r\left(h\right)}=\frac{4\cdot h^{2}+l^{2}}{8\cdot h}$$

When both ends are fixed or bent, the shape forms a hyperbolic cosine. The curvature and the radius of curvature are based on the radius of the largest circle that touches the center (Figure S12b, Supporting Information).

(7) $$2-\cos\left(h\left(x\right)\right)=1-\frac{x^{2}}{2!}-\frac{x^{4}}{4!}-\frac{x^{6}}{6!}-\frac{x^{8}}{8!}-\text{…}$$

Additionally, the Taylor expansions of 2−cos*(h)* and “cos” are completely identical up to the quadratic term of *x* Equation (7), which validates our approach. This method shows very little error when the curvature is small (i.e. when the radius of curvature is large). The error rate can be inferred from the data and was confirmed to be accurate within a 10% error margin. The experiment was conducted by pre‐calculating *h* to ensure that the curvatures had equal intervals.

### Preparation of Hydrogel Media

Soft and flexible media, including polyacrylamide hydrogel (PAA‐hydrogel) and a PDMS layer, were used between the ultrasound probe and the US‐TENG device. To prepare the PAA‐hydrogel, 14 g of acrylamide was dissolved in 160 mL of deionized water in a 250 mL glass beaker under magnetic stirring at room temperature. When the solution became clear, 0.03 g of lithium chloride, 0.8 g of MBAA, 0.29 g of potassium chloride, and 0.8 g of ammonium persulfate were added separately. The cross‐linking copolymerization of acrylamide and MBAA in aqueous solutions led to the formation of the PAA hydrogel. After mixing well and clear solution, 1.1 mL of TEMED was added as a curing agent to accelerate free radical formation from ammonium persulfate, initiating hydrogel formation. The solution was then cured at room temperature for 0.5 h.

### Device Simulation

The displacement of the vibrant PFA film (25 µm thickness, 3 × 3 cm^2^) in the ultrasound wave field was simulated using COMSOL Multiphysics. The simulation followed the experimental setup and material properties to predict displacement gradient values for modulated convex and concave shapes compared to the flat position. The distance in the underwater simulation was set at 3 and 6 cm for the flat shape, whereas in the hydrogel/PDMS medium, it was set at 3 cm for flat, concave, and convex shapes. The physical properties used in the simulation included:

PFA: Density = 2.15 g cm^−^^3^, Thickness = 25 µm, Young's Modulus = 498.4 MPa
PDMS: Density = 965 kg m^−^^3^, Thickness = 100 µm, Young's Modulus = 2.1 MPa
DI‐water: Density = 1 g cm^−^^3^, Thickness = 30 mm, Viscosity = 2.2 Pa·s
Hydrogel: Density = 1.2 g cm^−^^3^, Thickness = 29 mm, Young's Modulus = 6.78 kPa

### Electrical Circuit Design Of Battery

To evaluate wireless battery charging, a circuit was assembled on a breadboard with components for converting AC power from the US‐TENG_DF‐B_ to DC power for battery or capacitor charging. The circuit included a bridge rectifier with orthogonal junctions of four diodes (1N4007 PN) for AC‐to‐DC converting, a resistor (10kΩ) for capacitor discharge, rechargeable Lithium‐ion battery (Huahui New Energy, MH48909, 3.7 V, 90 mA.h), the capacitor (1000 µF, 25 V) for reducing voltage fluctuation, and a DC‐DC converter chip for making high‐efficiency output. Additionally, manual switchers were used for battery/capacitor disconnecting.

### Ex Vivo Setup for Changing the Battery

The performance of wireless battery charging was evaluated using ex vivo soft tissue mimics. A pork belly with fat, muscle, and skin layers was cut 10 × 10 cm^2^ pieces with varying thicknesses. The PDMS‐covered US‐TENG_DF‐B_ was implanted within the tissue at a specific probe‐device distance. An ultrasound probe was placed on the skin over the implanted device. To test device feasibility in curved positions, the device was mounted on printed semicircular supports with diameters of 3.2, 4.5, and 6.4 cm, corresponding to different bending degrees, calculated based on the circumference‐to‐diameter ratio (c/d = π).

## Conflict of Interest

The authors declare no conflict of interest.

## Author Contributions

I.M.I. and H.S.K. contributed equally to this work. This project's experiments and methodology design were originally conceptualized and visualized by I.M.I. and H.S.K.; the provided extensive experiments such as material characterizations on the measurements were suggested by M.L., S.‐B.K., S.‐M.S. and D.‐G.L.; the simulation process was proceeded by S. K. Some materials were provided by J.‐H.H., J.L. and I.‐Y.S. All the authors discussed the results and commented on the revision and refinement of the manuscript, and I.M.I. wrote the manuscript; S.‐W.K., H.K., D.S. and J.M.B. supported the investigation process; S.H. and H.‐C.S. (supervisor and corresponding) led to providing financial support and oversight of the whole project validation.

## Supporting information



Supporting Information

Supplemental Video 1

Supplemental Video 2

## Data Availability

The data that support the findings of this study are available from the corresponding author upon reasonable request.
