# Supplementary material for: A Body Conformal Ultrasound Receiver for Efficient and Stable Wireless Power Transfer in Deep Percutaneous Charging
Source: Adv Mater. 2025 Mar 26;37(19):2419264. doi: 10.1002/adma.202419264 (PMC12075921; doi:10.1002/adma.202419264)
Supplement: Supplementary file 1 — Supporting Information [file ADMA-37-2419264-s001.docx]

Supporting Information

A Body Conformal Ultrasound Receiver for Efficient and Stable Wireless Power Transfer in Deep Percutaneous Charging

*Iman M. Imani, Hyun Soo Kim, Minhyuk Lee, Seung-Bum Kim, So-Min Song, Dong-Gyu Lee, Joon-Ha Hwang, Jeyeon Lee, In-Yong Suh, Sang-Woo Kim, Jun Chen, Heemin Kang, Donghee Son, Jeong Min Baik, Sunghoon Hur*, Hyun-Cheol Song**

**Contents:**

**⏺ Supporting figures:**

Figure S1: The fabrication process of the US-TENG_DF-B_ device step by step.

Figure S2: Schematic diagrams of the different US-TENG devices with different components for advantage comparison.

Figure S3: Study of the electrical properties of P(VDF-TrFE)/CCTO composite films.

Figure S4: Polarization hysteresis loops of the polarized P(VDF-TrFE)/CCTO composite with different concentrations.

Figure S5: Chemical structure identification by FT-IR spectroscopy spectra.

Figure S6: The comparisons of the surface potential of PFA, PTFE, Nylon 6,6, and PU films were measured by Kelvin probe force microscopy (KPFM).

Figure S7: Schematically information for ferroelectricity of poled/non-poled P(VDF-TrFE).

Figure S8: Chemical structure identification by XRD pattern for CCTO powder, P(VDF-TrFE), and poled/non-poled P(VDF-TrFE)/CCTO composite

Figure S9: SEM images of CCTO particles.

Figure S10: Plotted the roughness and surface potential difference values of various P(VDF-TrFE)/CCTO composites with polarization and non-polarization stats.

Figure S11: The comparison of KPFM images with surface potential difference values of P(VDF-TrFE)/CCTO composites with polarization and non-polarization states, and the comparison of AFM images with roughness difference values of P(VDF-TrFE)/CCTO composites with polarization and non-polarization states.

Figure S12: Modulation of the bending response of the flexible device for correcting geometric measurements.

Figure S13: Experimental setups of underwater and ex vivo tests for evaluating the output performance and battery charging of ultrasound-driven PDMS-covered US-TENG_DF-B_ device inside DI-water and porcine tissue.

Figure S14: The rectified DC voltage output graph at different ultrasound intensities under ex vivo conditions.

Figure S15: Output performance results of the deformed device under porcine tissue.

Figure S16: The result of charge density generated by the US-TENG_DF-B_ under variant conditions.

Figure S17: The results of stable electrical output over multiple convex-concave bending cycles in underwater conditions, confirm the device’s mechanical resilience.

Figure S18: Biocompatibility results of PDMS-capsulated US-TENG_DF-B_ device through MTT assay after 1, 2, and 3 days with CRL-1502 cells.

**⏺** **Supporting Table:**

Table S1: Power and size information of the commercial battery-required implantable electronic devices.

Table S2: Comparisons of features and durability of the last reported trend technology on wireless battery charging and our present work.

**⏺** **Captions for the Supporting Videos:**

Video S1: The oscilloscope screened voltage signal output by contact-separation of pushing test for comparing the performance of the tribo-layers

Video S2: DC and AC energy harvesting from US-TENG_DF-B_ device under porcine tissue and underwater respectively.

**
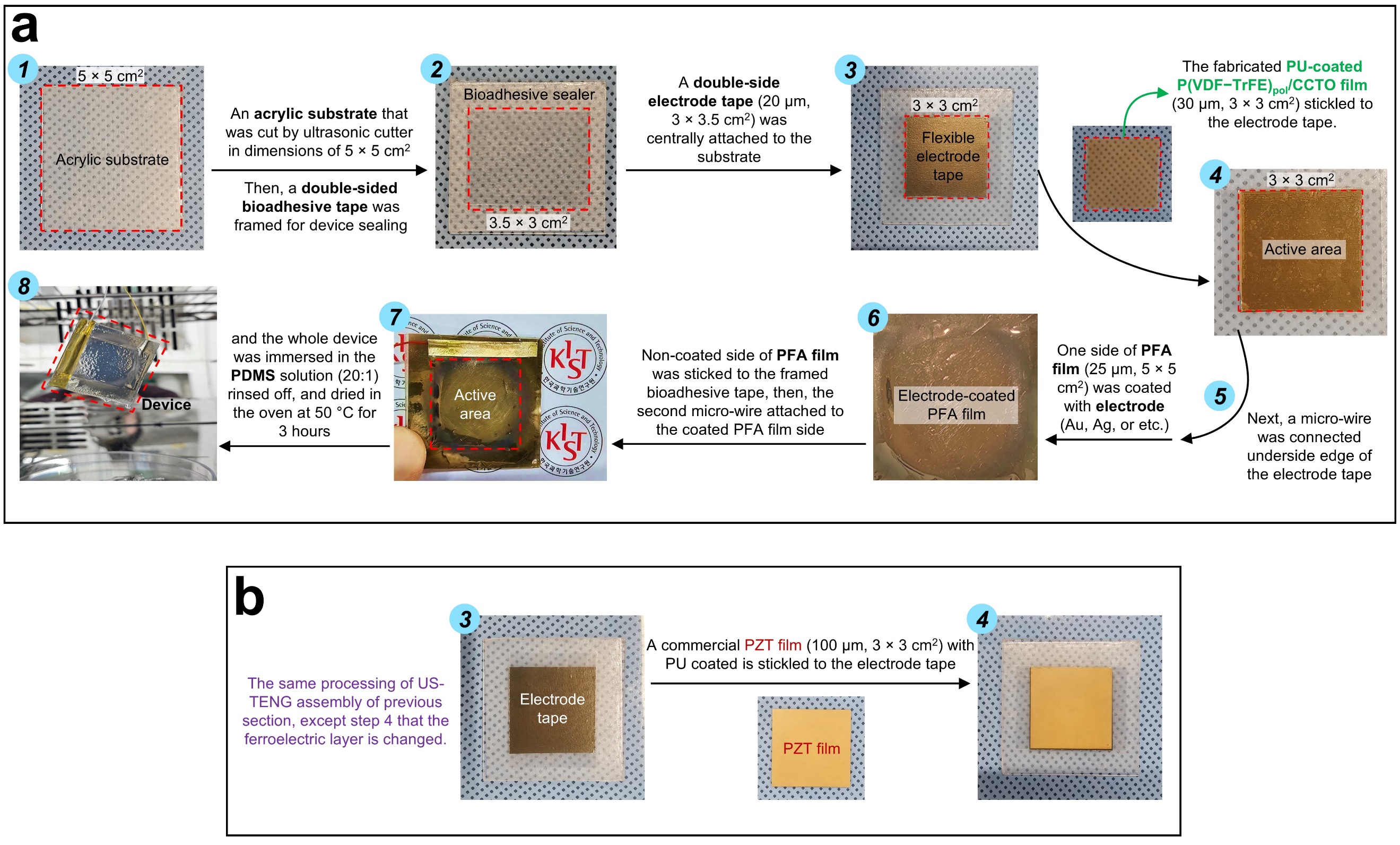
**

**Figure S1:** The fabrication process of the US-TENG_DF-B_ device step by step. **a,** and **b,** fabricating the US-TENG including a substrate film of flexible and inflexible PMMA with 0.1 mm and 1.2 mm thicknesses respectively.


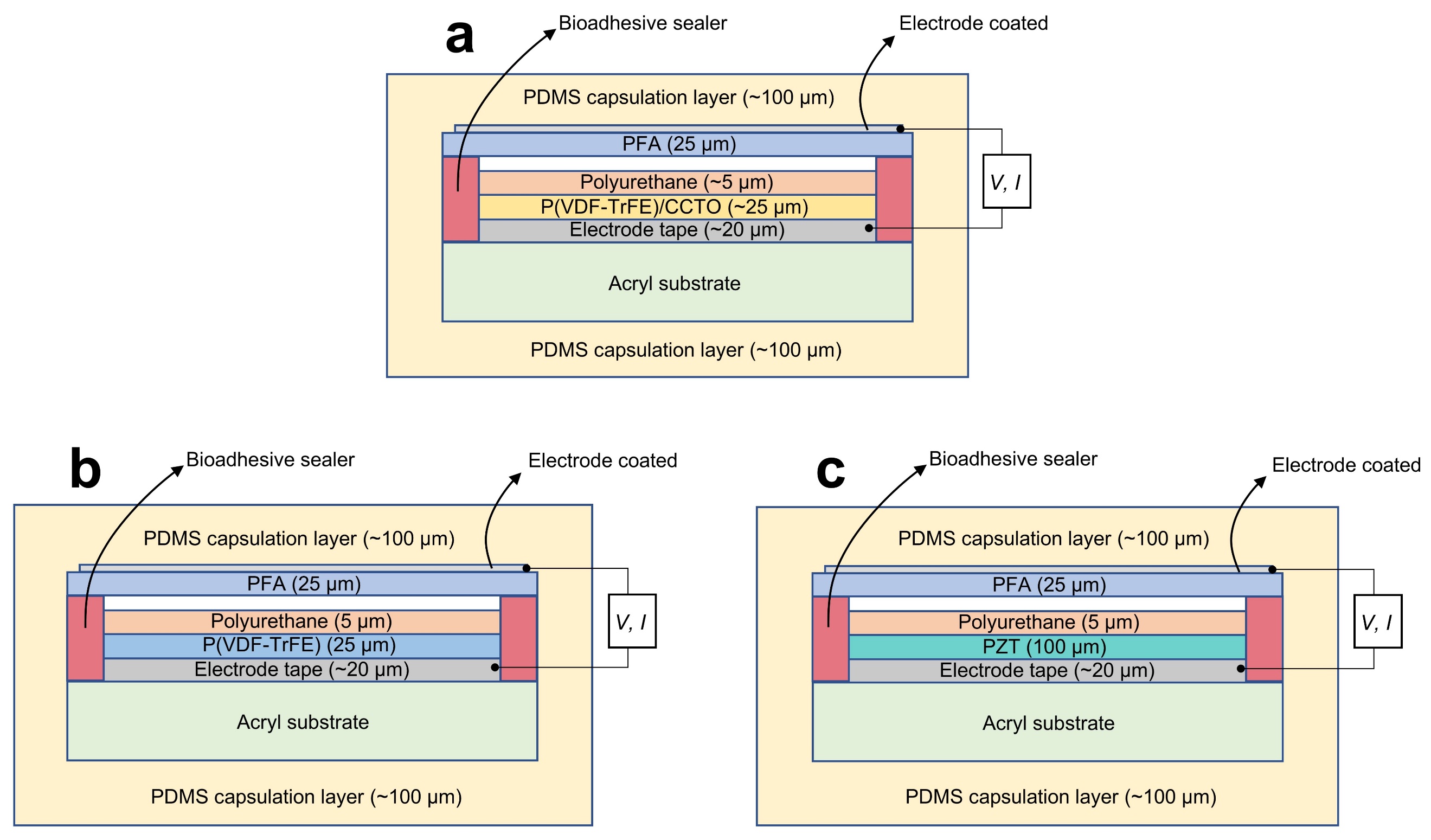


**Figure S2:** Schematic diagrams of the different US-TENG devices with varying components for advantage comparison. **a.** The materials structure of the US-TENG_DF-B_ device boosted by P(VDF-TrFE)/CCTO (this work), **b.** the materials structure of the US-TENG device boosted by P(VDF-TrFE), **c.** the materials structure of the US-TENG boosted by PZT.

**
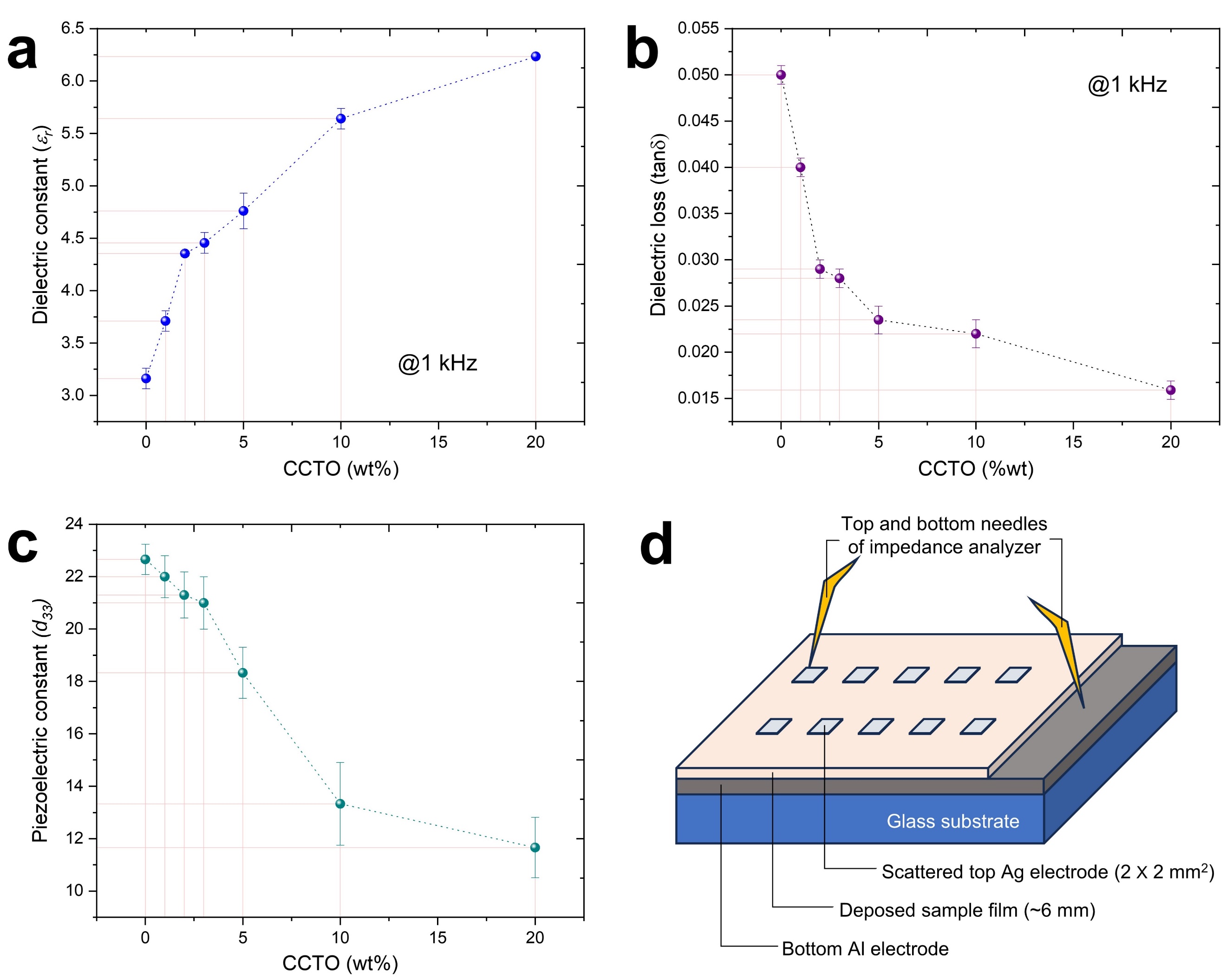
**

**Figure S3:** Study of the electrical properties of P(VDF-TrFE)/CCTO composite films. Frequency-dependent, **a.** dielectric constant, **b.** tangent loss, and **c.** piezoelectric constant respectively. **d.** Components of the fabricated chip for analyzing impedance properties of the samples.


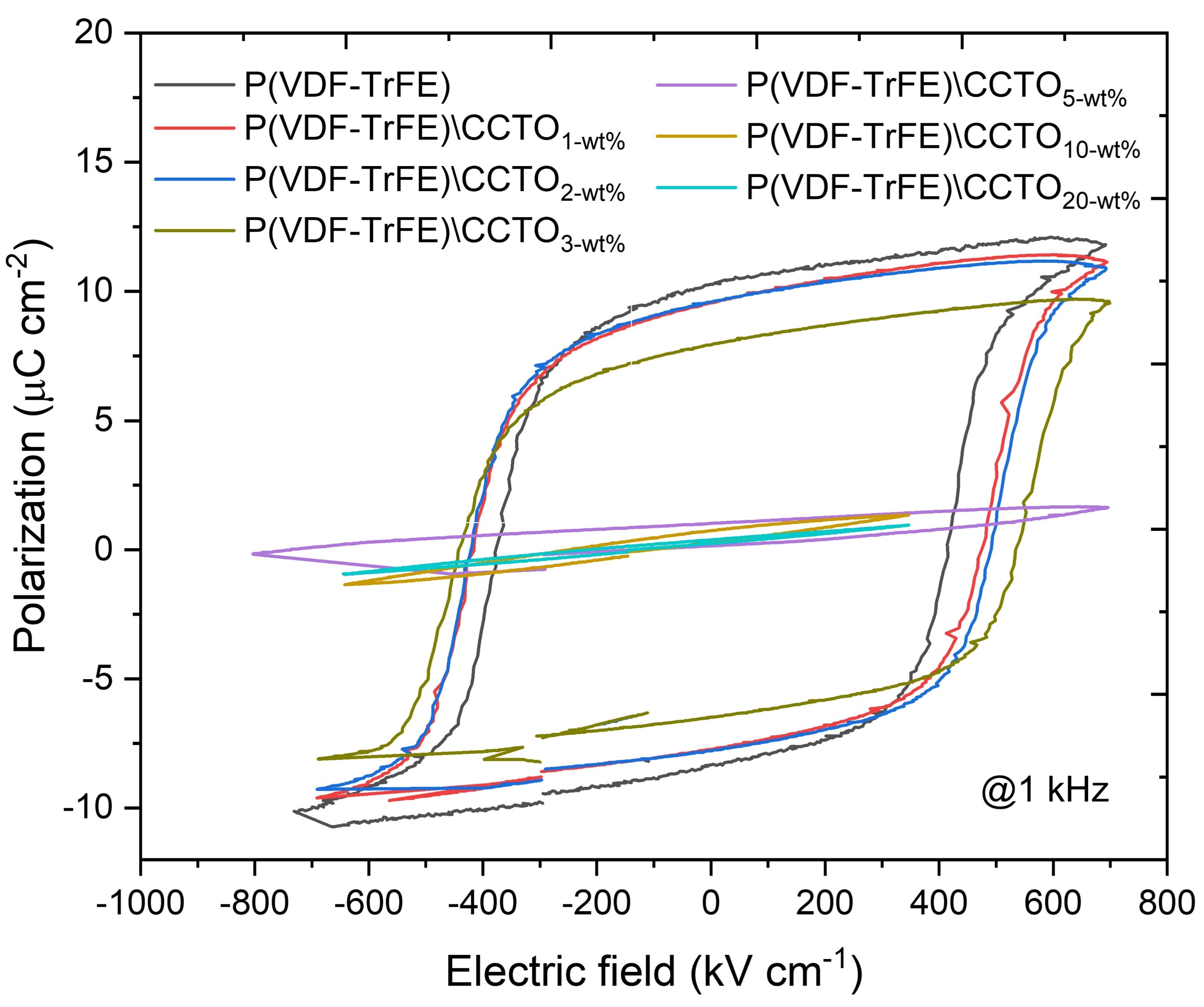


**Figure S4:** Polarization hysteresis loops of the polarized P(VDF-TrFE)/CCTO composite with different concentrations.


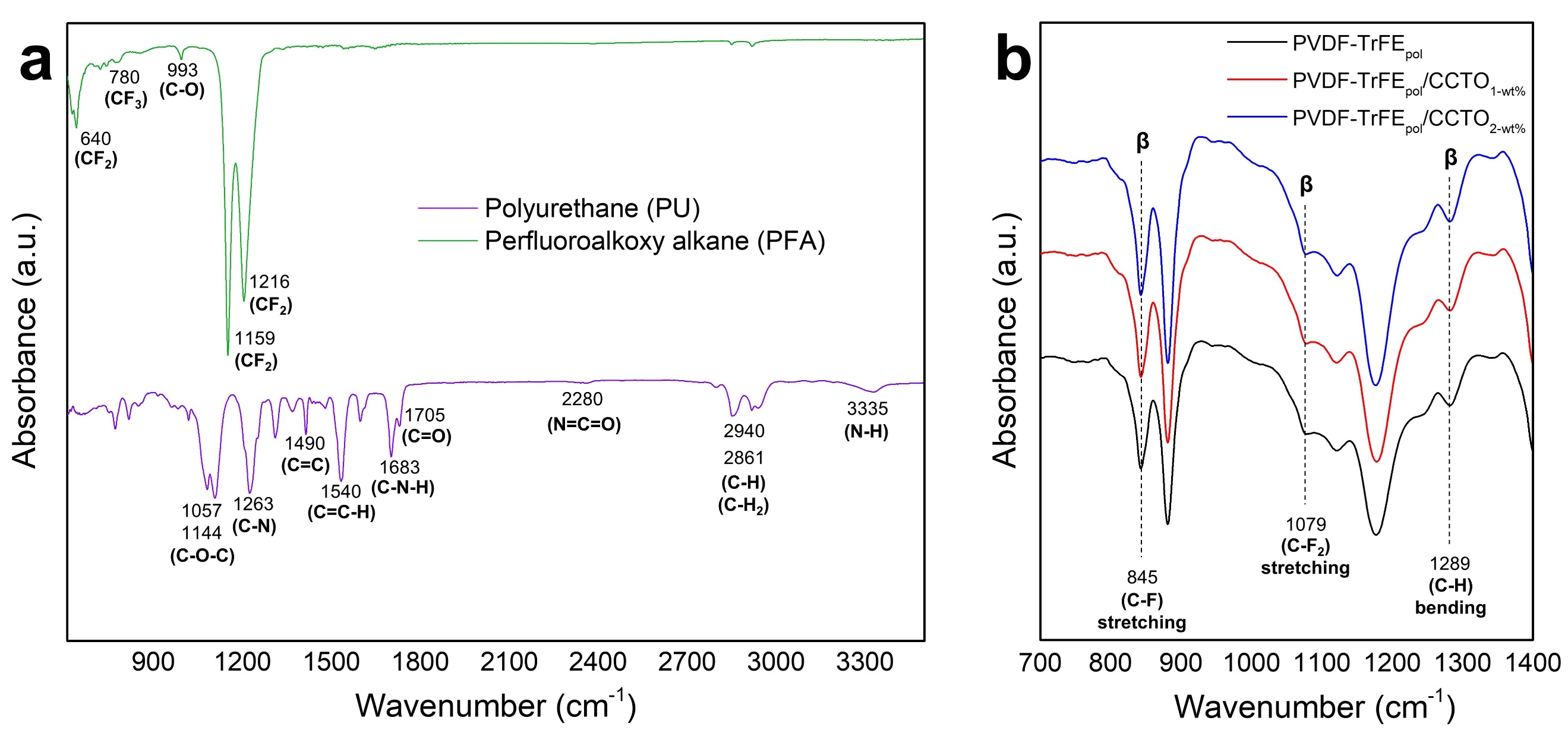


**Figure S5:** Chemical structure identification by FT-IR spectroscopy spectra. **a.** confirmation of the used triboelectric polymer films (PFA and PU), **b.** confirmation of fabricated poled P(VDF-TrFE)/CCTO composites.


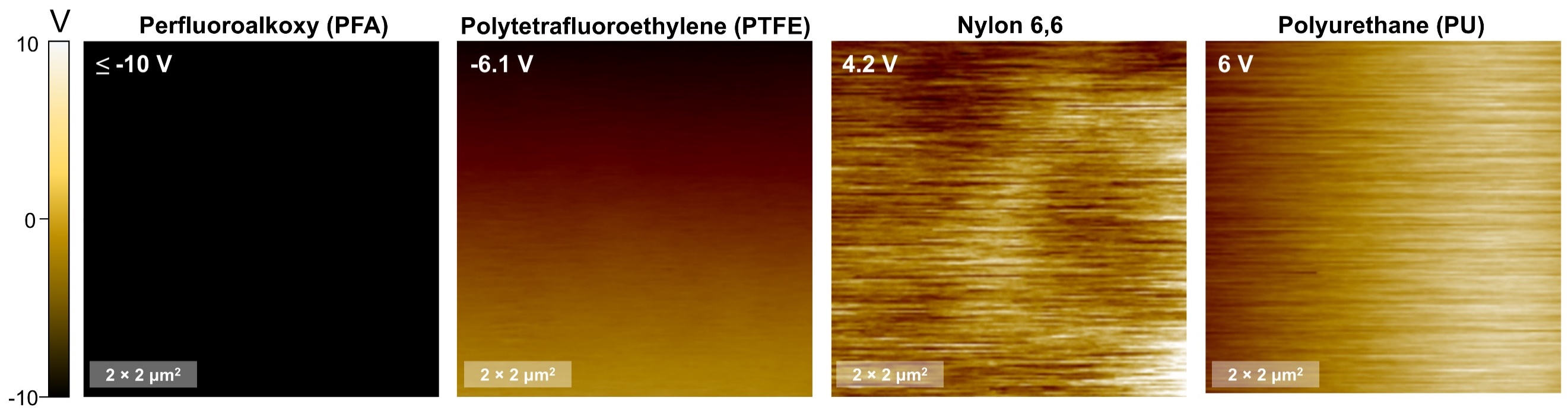


**Figure S6:** The comparisons of the surface potential of PFA, PTFE, Nylon 6,6, and PU films were measured by Kelvin probe force microscopy (KPFM).


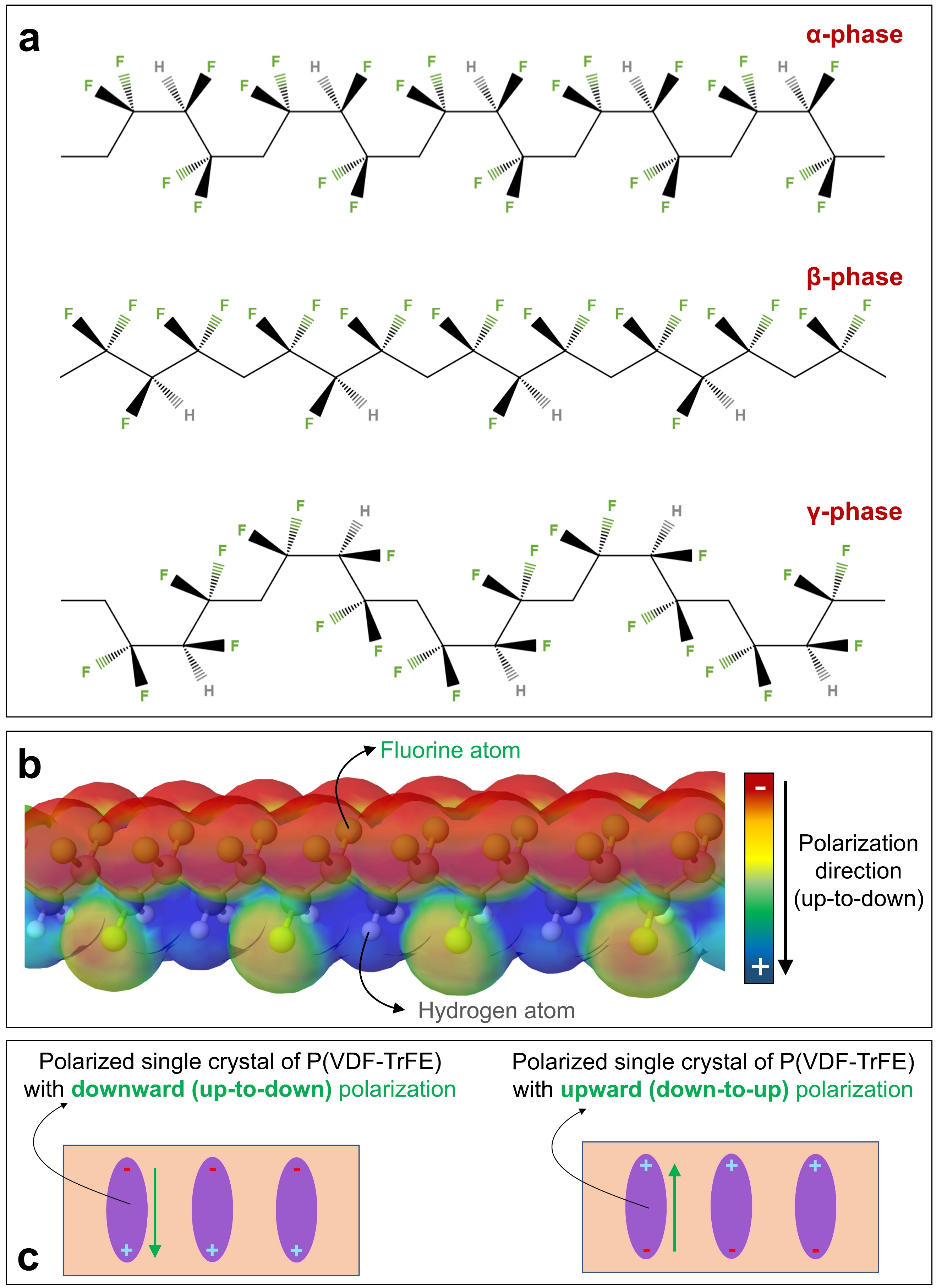


**Figure S7:** Schematically information for ferroelectricity of poled/non-poled P(VDF-TrFE). **a.** Chemical Structure of P(VDF‐TrFE) with different phases. **b.** Molecular electrostatic potential maps showing electron density of β-P(VDF-TrFE). **c.** Polarization directions of single crystals of β-P(VDF-TrFE).


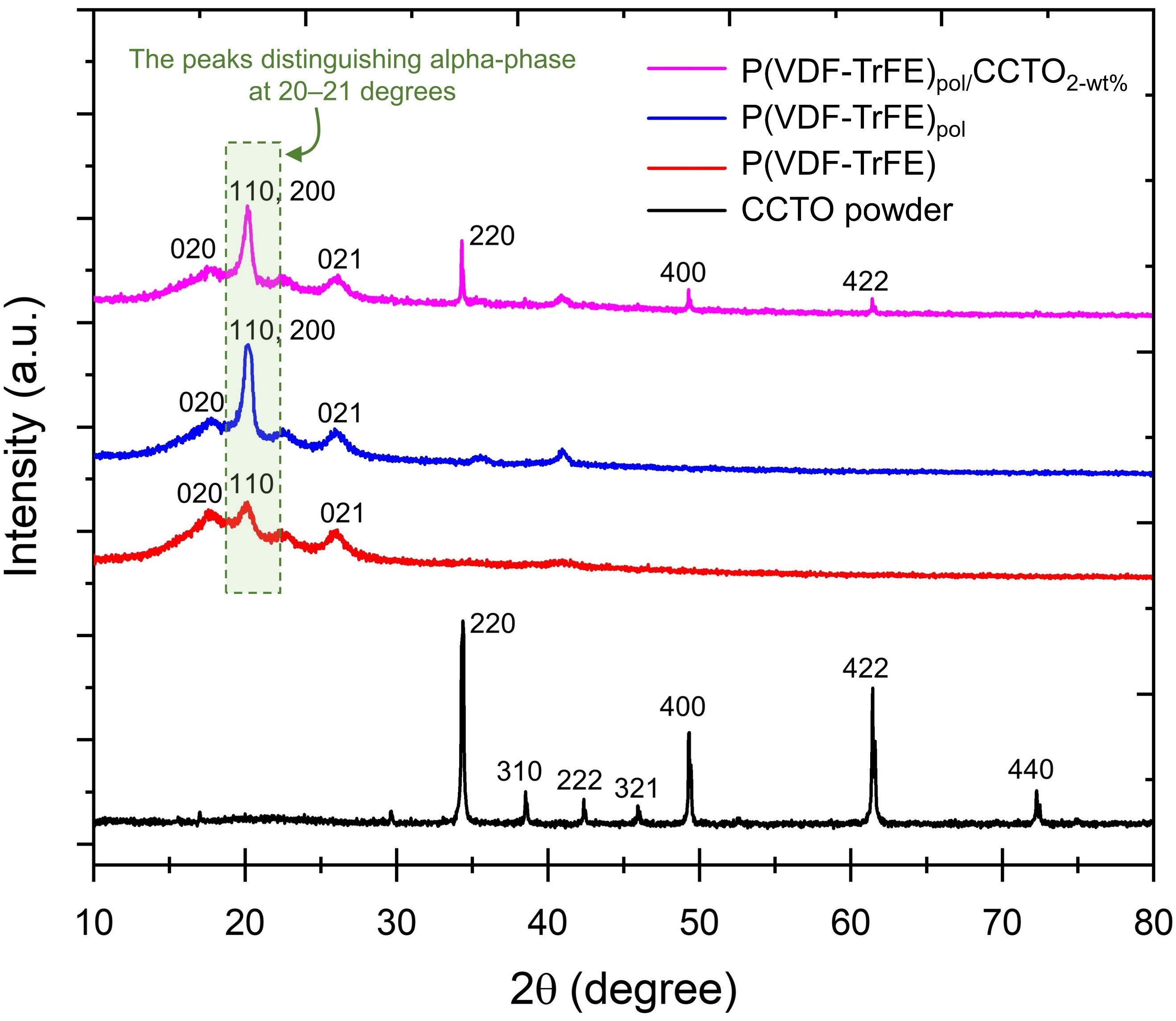


**Figure S8:** Chemical structure identification by XRD pattern for CCTO powder, P(VDF-TrFE), and poled/non-poled P(VDF-TrFE)/CCTO composite.


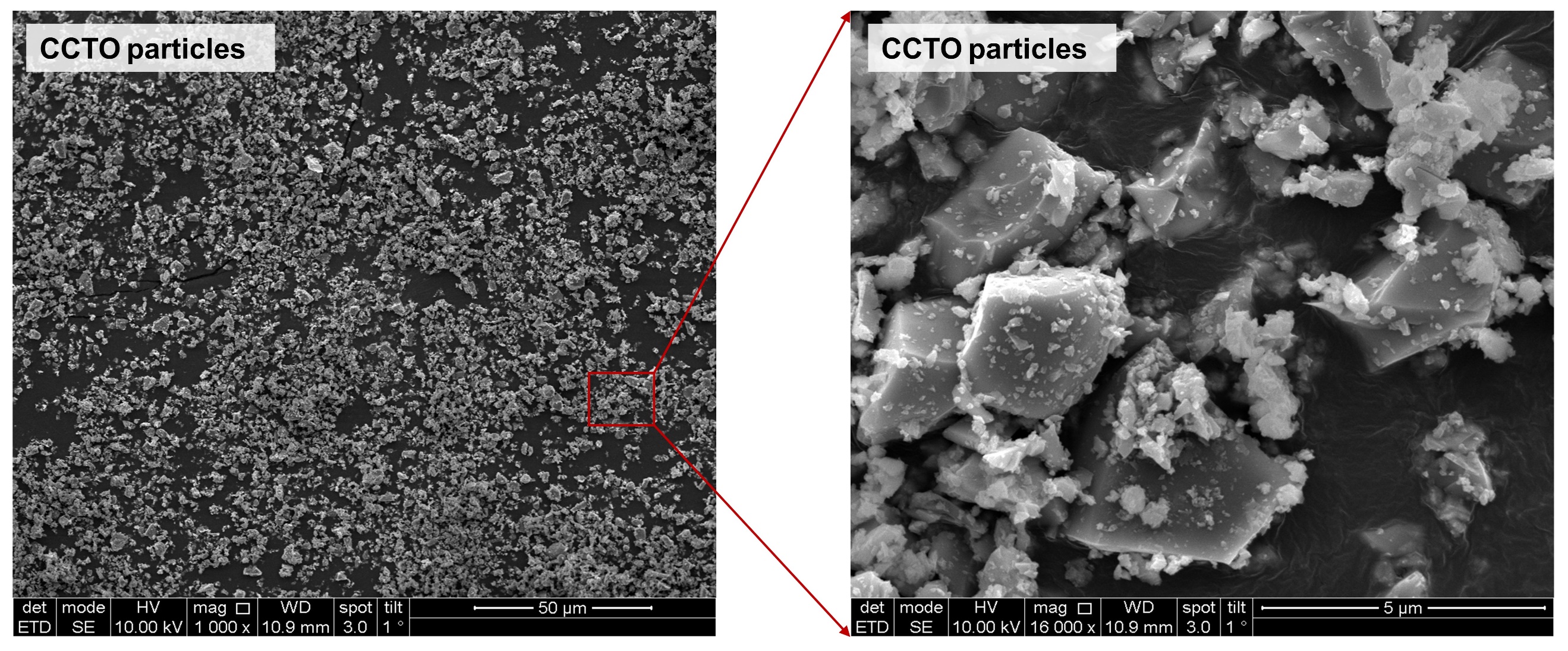


**Figure S9:** SEM images of CCTO particles.


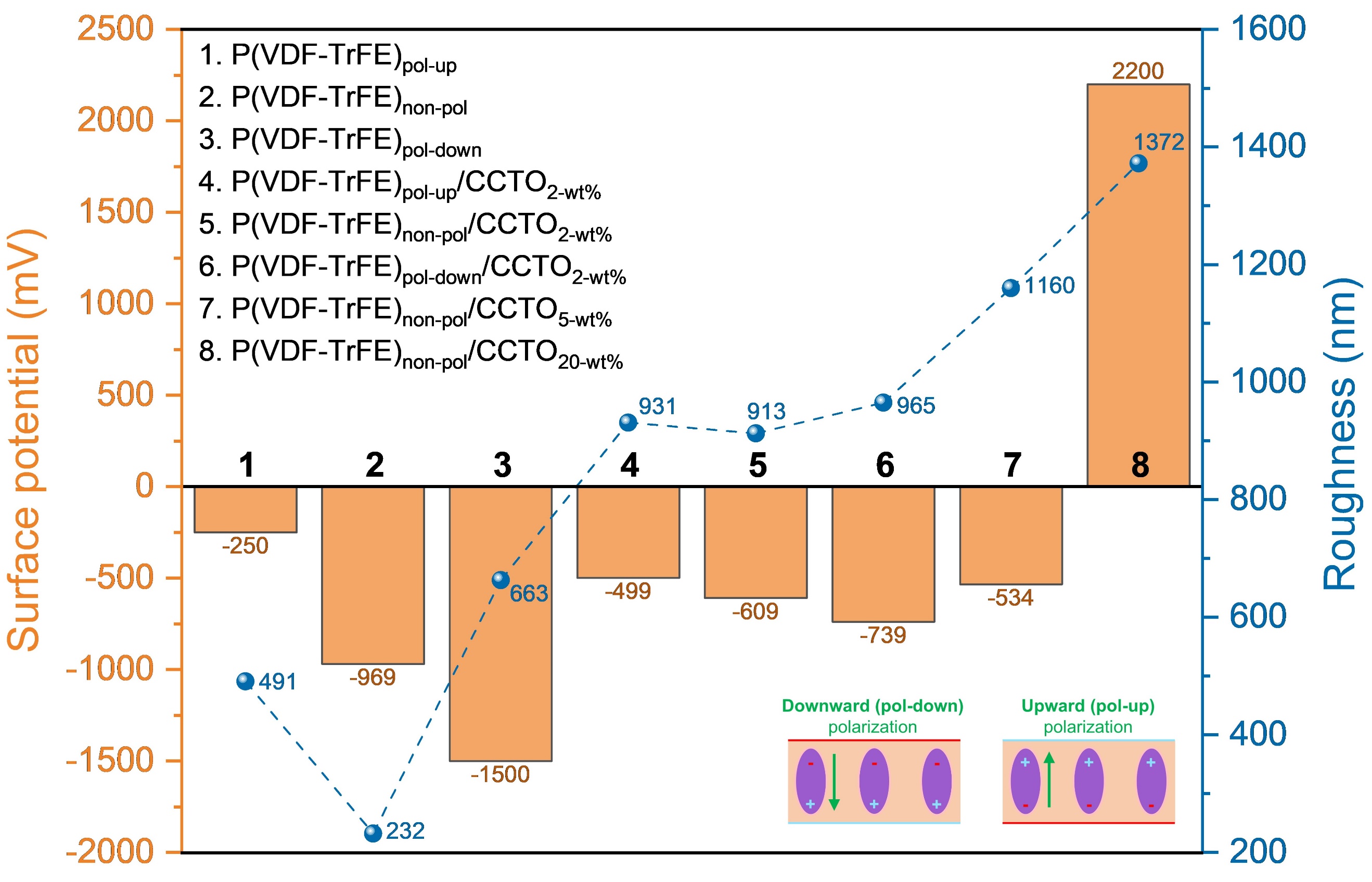


**Figure S10:** Plotted the roughness and surface potential difference values of various P(VDF-TrFE)/CCTO composites with polarization and non-polarization stats.


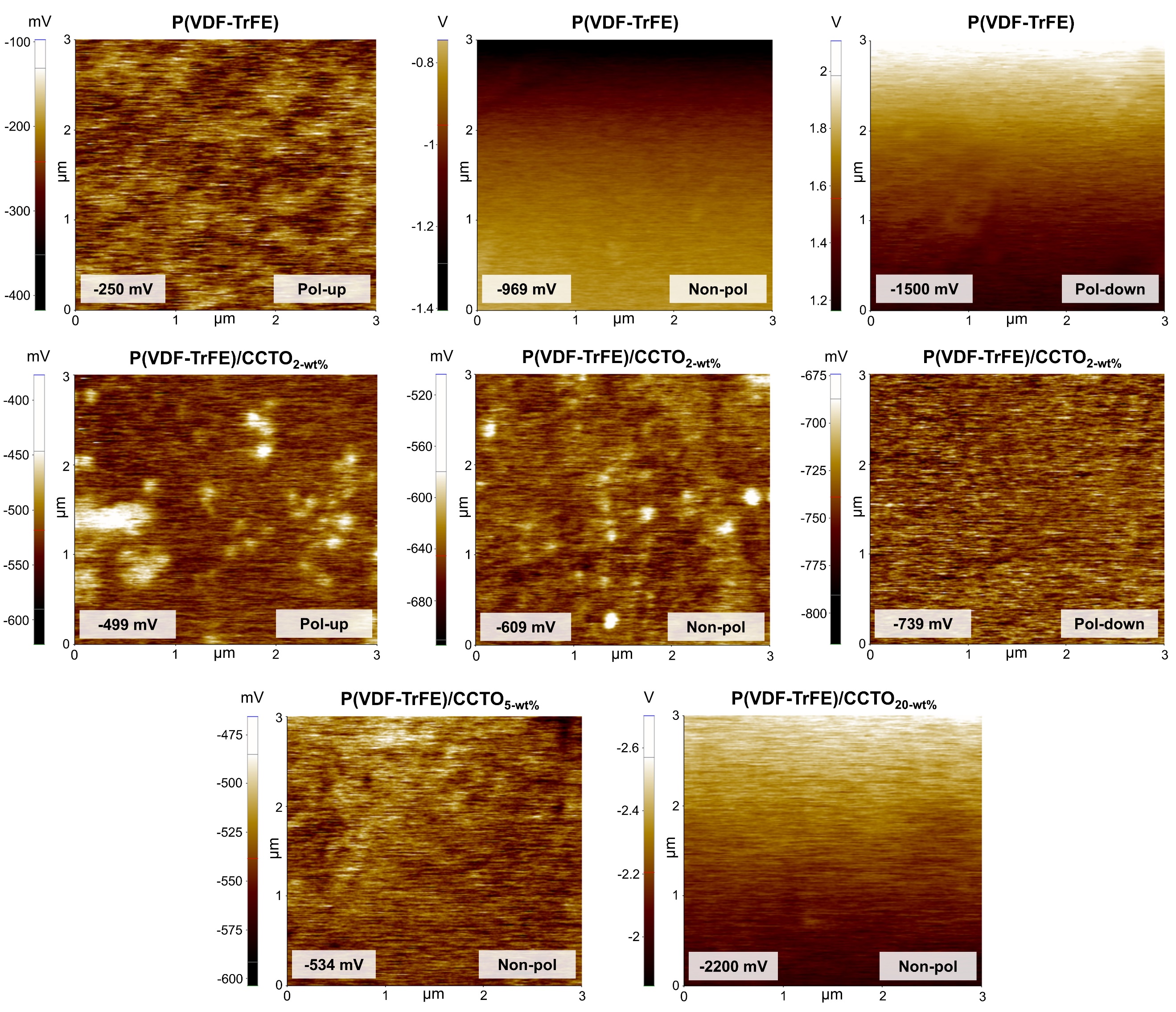


**a**

**b**

**
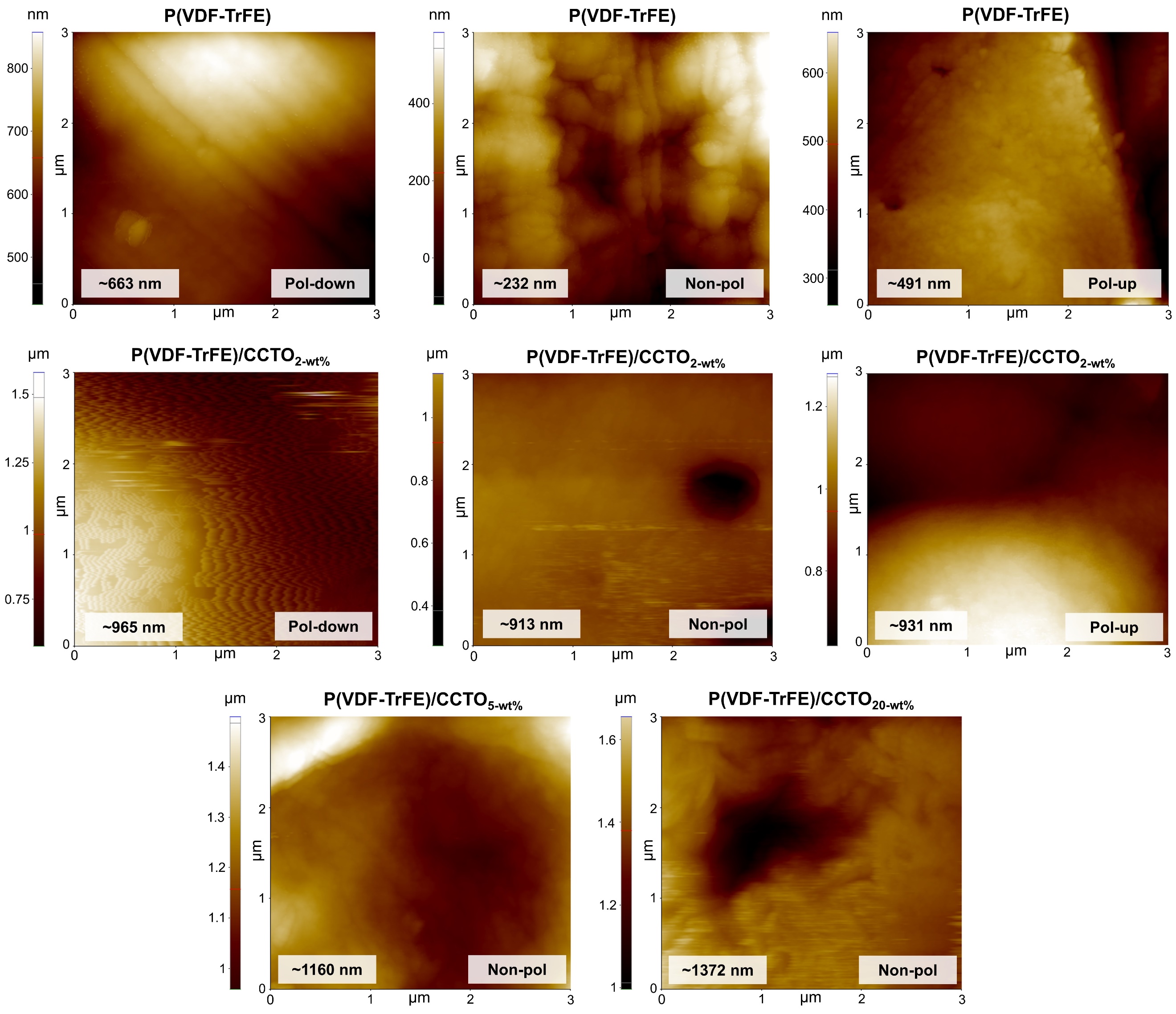
**

**Figure S11:** **a.** The comparison of KPFM images with surface potential difference values of P(VDF-TrFE)/CCTO composites with polarization and non-polarization states. **b.** The comparison of AFM images with roughness difference values of P(VDF-TrFE)/CCTO composites with polarization and non-polarization states.


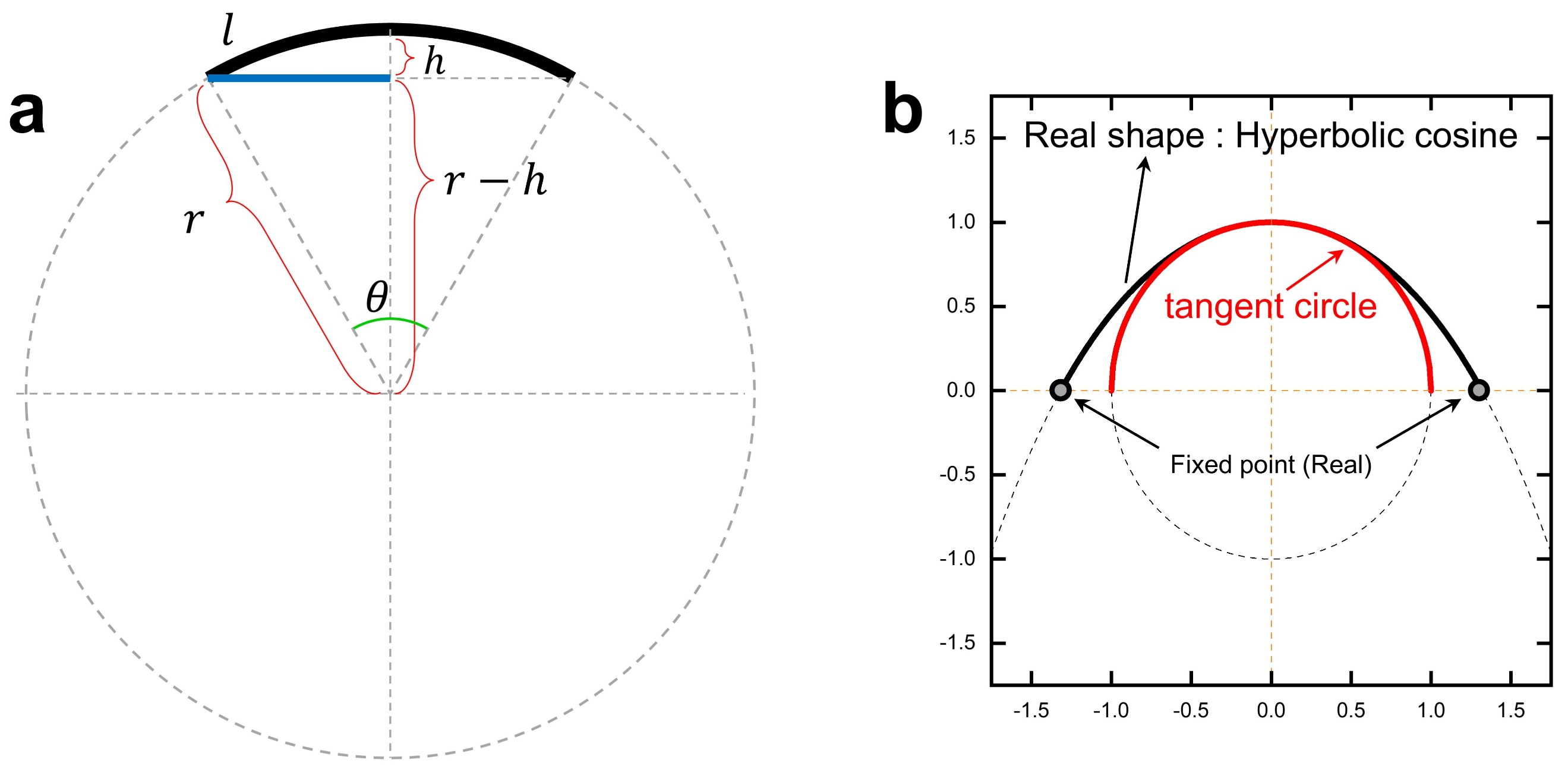


**Figure S12:** Modulation of the bending response of the flexible device for correcting geometric measurements. **a.** The hypothetical circle’s arc/radius (*l, r*) and angle of bending (*θ*) on the curved surface of the device. **b.** The shape forms a hyperbolic cosine of both ends fixed and bent.


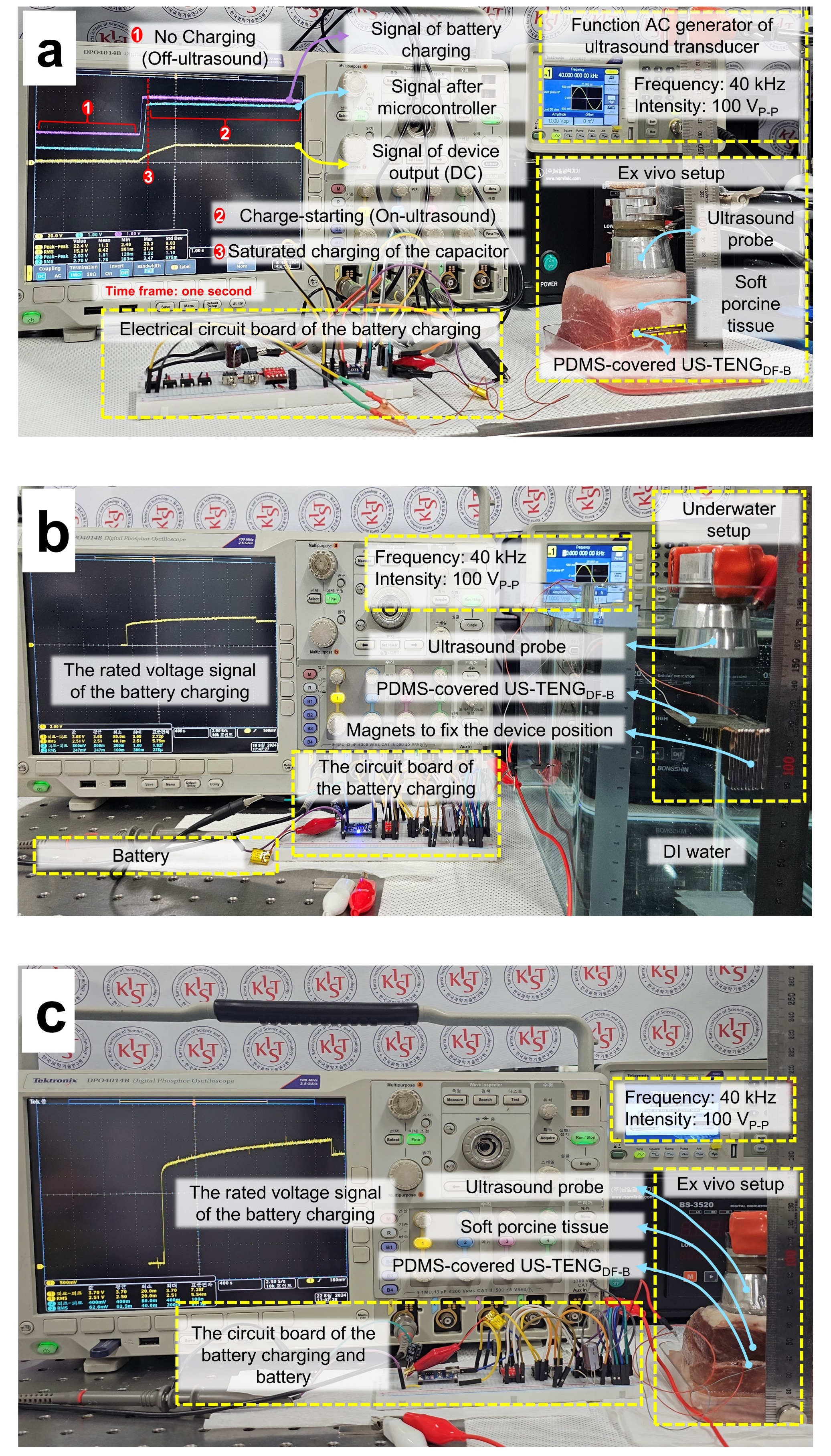


**Figure S13:** Experimental setups of **a.** ex vivo output performance through DC open circuit of battery, **b.** and **c.** battery charging of ultrasound-driven PDMS-covered US-TENG_DF-B_ device inside DI-water and porcine tissue.


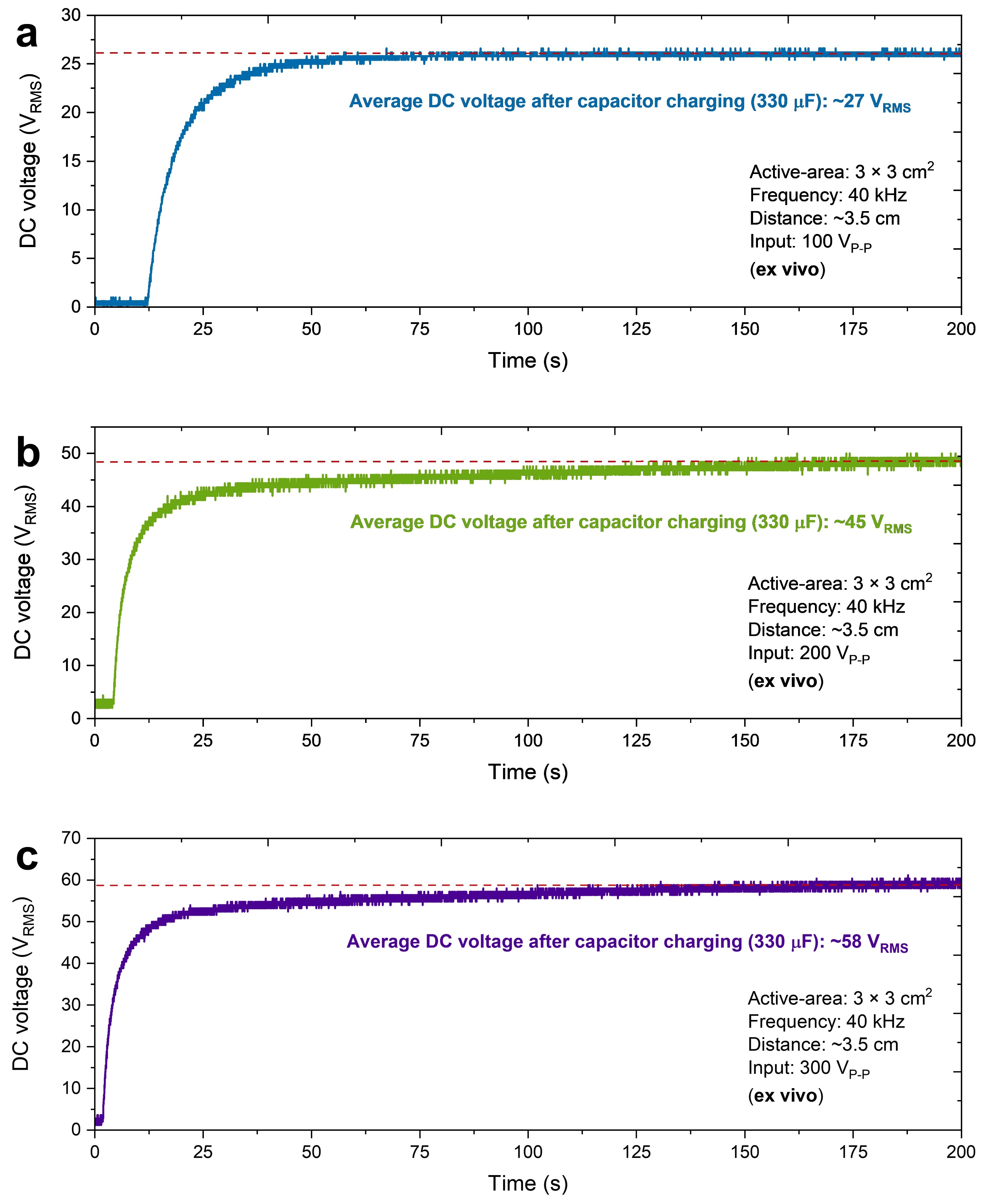


**Figure S14:** The rectified DC voltage output graph at different ultrasound intensities under ex vivo conditions.


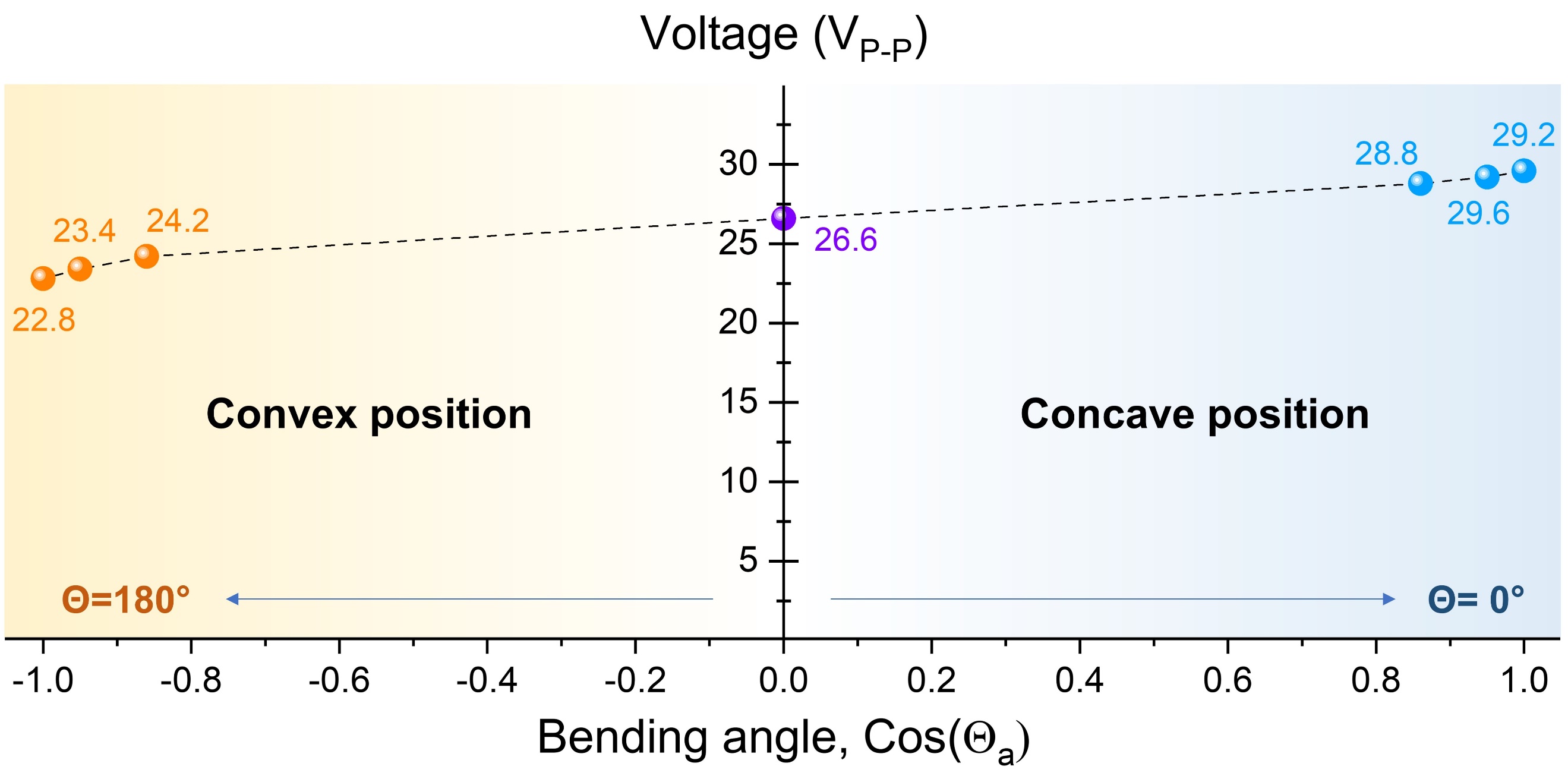


**Figure S15:** Output performance results of deformed device under porcine tissue. a. Peak-to-peak voltage amplitudes of ultrasound-driven device embedded under porcine tissue (~35 mm distance, 100 V_P-P_ input, 40 k Hz) for different curvature grades of device-attached cantilevers.

**
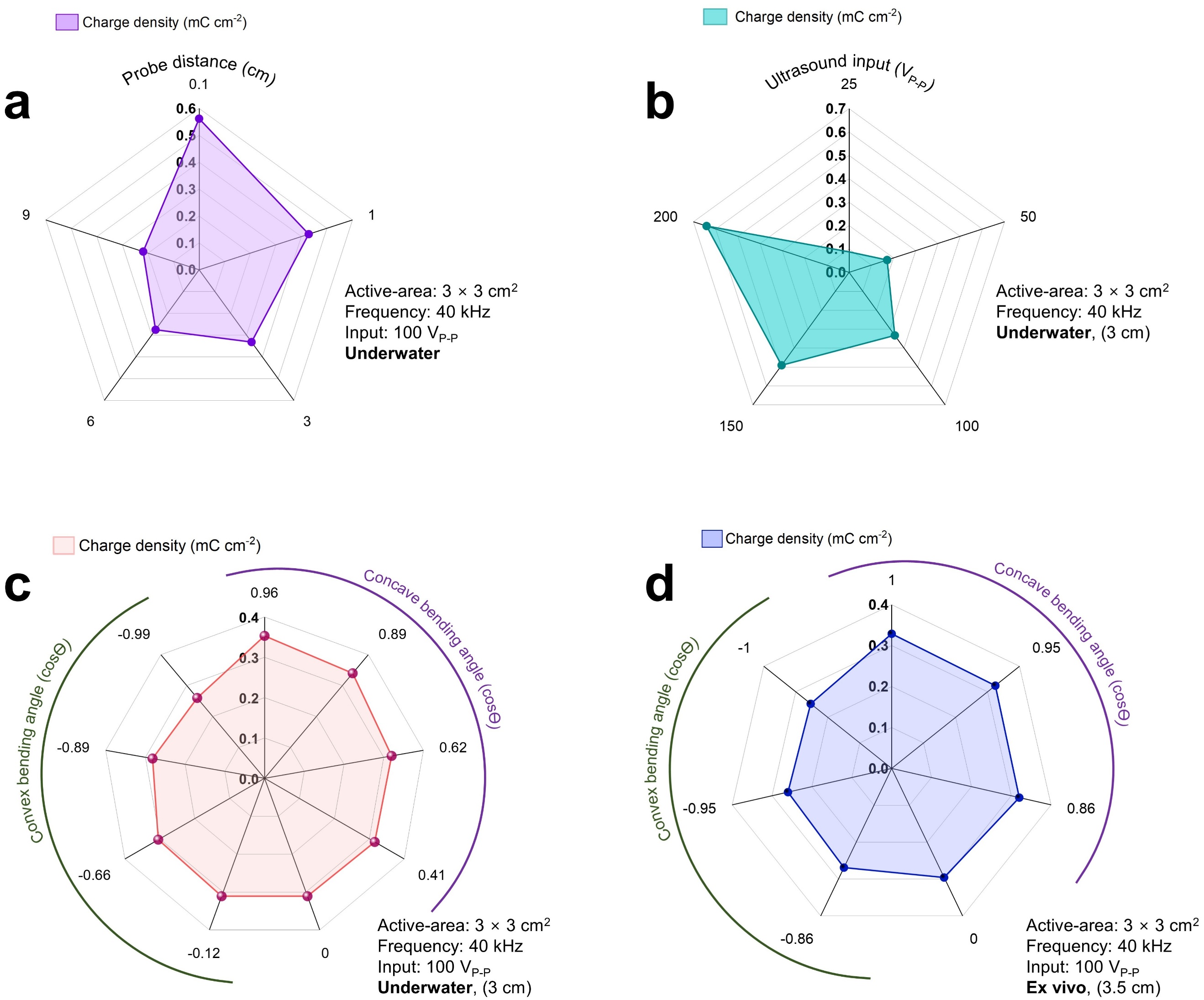
**

**Figure S16:** The result of charge density generated by the US-TENG_DF-B_ under variant conditions: **a. and b.** different probe distances and ultrasound intensities underwater, **c.** different convex-concave bending of the device underwater, and **d.** different convex-concave bending of the device in ex vivo.

**
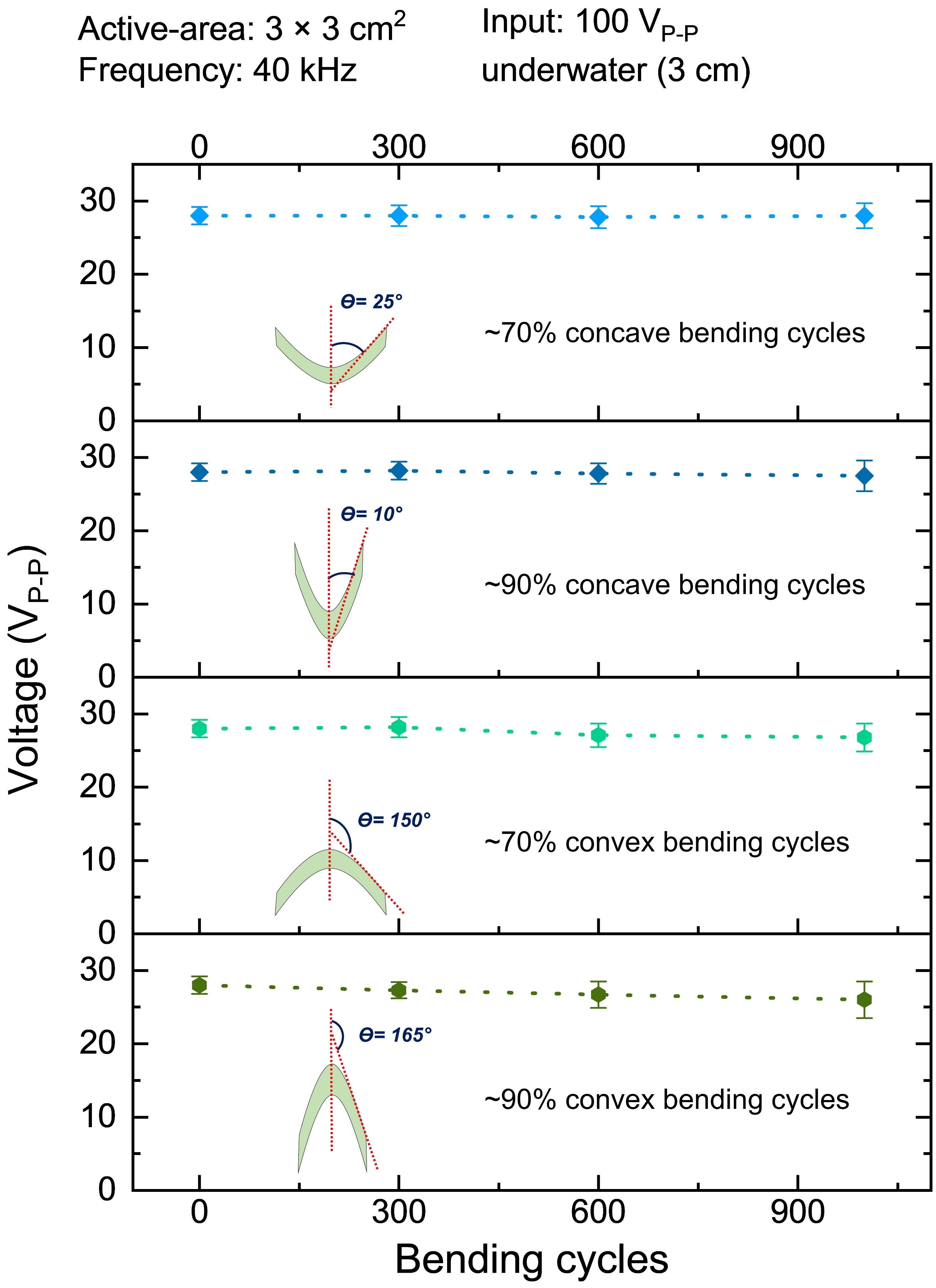
**

**Figure S17:** The results of stable electrical output over multiple convex-concave bending cycles in underwater conditions, confirm the device’s mechanical resilience.


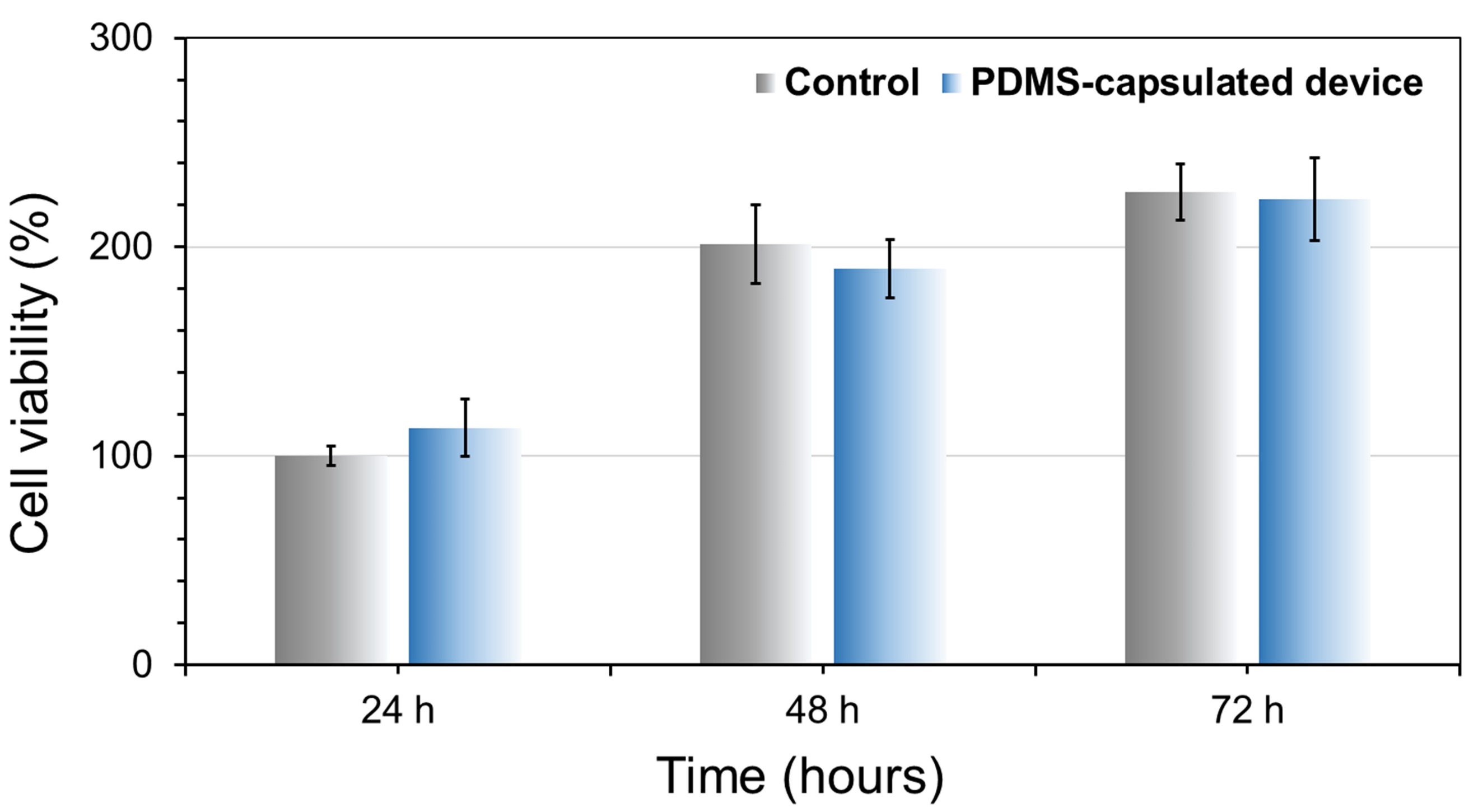


**Figure S18:** Biocompatibility results of PDMS-capsulated US-TENG_DF-B_ device through MTT assay after 1, 2, and 3 days with CRL-1502 cells.

**Table S1:** Power and size information of the commercial battery-required implantable electronic devices.

| **Name of implantable electronic device** | **Application** | **Battery capacity**  **(Current amplitude)** | **Rechargeability and lifetime of the device** | **Size of device** |
| --- | --- | --- | --- | --- |
| directSTIM™ DBS  (ALEVA) | DBS | Current amplitude: a maximum total of 30 mA | Rechargeable battery by MEMS | 4 cm × 6 cm |
| ACCOLADE-ESSENTIO MRI pacemakers  (Boston Scientific) | Pacing and automatic daily monitoring | 1.6 ampere-hour | Lifetime of ~13 years | - |
| VIGILANT^TM^ EL,  RESONATE™ HF,  MOMENTUM™ EL,  (Boston Scientific) | ICD | 1.9 ampere-hour  Pacing amplitude: 2.5 V | Lifetime of ~9 years | 5.37 × 7.79 × 0.99 cm^3^ |
| VISIONIST™,  VALITUDE™  (Boston Scientific) | Cardiac resynchronization therapy pacemakers (CRT-P) | 1.6 ampere-hour | Lifetime of ~10 years | - |
| INOGEN™ X4  (Boston Scientific) | Cardiac resynchronization therapy defibrillator (CRT-D) | 1.9 ampere-hour  Pacing amplitude: 2.5-5 V | Lifetime of ~2 years | 5.37 × 8.18 × 0.99 cm^3^ |
| Precision Montage™ MRI  (Boston Scientific) | Spinal cord stimulator system | Current amplitude: a maximum total of 25.5 mA | Rechargeable | 19.8 cm^3^ |
| Nucleus®  Cochlear | Bone-conduction hearing implants for stimulation of the cochlea | Current amplitude: a maximum total of 1.75 mA | Rechargeable | 5.05 × 3.1 × 0.39 cm^3^ |
| SYNCHRONY | Cochlear implant,  bone-conduction hearing implants for stimulation of the cochlea | Current amplitude: a maximum total of 1.2 mA | Rechargeable | 4.57 × 2.54 × 0.45 cm^3^ |
| SynchroMed™ II | implantable programmable drug pump delivery  (intrathecal pump) | Current amplitude: a maximum total of 12.5 mA | Rechargeable  The lifetime of 6-7 years | Thickness: 26mm  Diameter: 87.5mm |
| EBI OsteoGen^TM^  (ZimVie) | Bone growth stimulation | A minimum total of 0.2 A | - | 36 × 23 × 5 mm^3^ |
| RestoreAdvanced,  Activa PC,  Activa RC  (Medtronic) | Spinal cord neurostimulator | Amplitude: a maximum total of 10.5 V and 25.5 mA | Rechargeable  Depends on settings and use | 65 × 49 × 15 mm^3^ |
| EMBLEM™ MRI  (Boston Scientific) | ICD | - | Lifetime of ~6 years | 83.1 × 69.1 × 12.7 cm^3^ |
| LUX-Dx  (Boston Scientific) | Insertable cardiac monitor | - | Battery lifetime of ~3 years | 1.2 cm^3^ |
| aura6000™ (LivaNova) | Hypoglossal nerve stimulator for optimizing obstructive sleep apnea | - | Rechargeable | - |
| SYMMETRY™  (LivaNova) | Vagus nerve stimulation therapy for difficult-to-treat depression | - | Rechargeable | - |
| Enterra™  (Medtronic) | Implantable programmable gastric neurostimulator | Amplitude: a maximum total of 10.5 V | - | 5.5 × 6 × 1.14 cm^3^ |

**Table S2:** Comparisons of features and durability of the last reported trend wireless battery charging technologies based on piezoelectric/triboelectric generators and our present work.

| **Latest Wireless Charging Devices Presented** | **Application** | **Output Power** | **Ultrasound frequency and intensity** | **Implanted device Size** | **Implant Depth & Misalignment** **with charging ability** | **Flexibility** | **Ref** |
| --- | --- | --- | --- | --- | --- | --- | --- |
| Electret films of lead-free piezoceramics  (Flat structure) | Charging implantable electronic devices | 14 V and 12.9 μW at 5 cm  (ex vivo) | 100 kHz,  200 V | (3 × 3 × 1 mm^3^) × 4 pieces of the active area | Up to 2.5 cm depth | Yes | ^61^  (2023) |
| Ferroelectric crystal composite of Nd-PCC  (Helix-like structure) | Charging implantable electronic devices | 15.6 V and 22 μW at 2.5 cm  (ex vivo) | 500 kHz,  0.3 W cm^-2^ | (1 × 1 × 0.3 mm^3^) × 24 pieces of the active area | Up to 2.5 cm depth with different rotating angles up to ~60° | Yes | ^17^  (2024) |
| US-TENG  (multilayer structure) | Charging implantable electronic devices | 50 V, 1 mA, and 6.4 mW at 0.5 cm  (ex vivo) | 20 kHz,  1 & 3 W cm^-2^ | 36 × 36 mm^2^ of the active area | Up to 1 cm depth | No | ^39^  (2019) |
| Ferroelectrically augmented US-TENG  (multilayer structure) | Charging implantable electronic devices | ~5 V_P-P_ at 3 cm  (ex vivo) | 40 kHz,  0.2 W cm^−2^  100 V_P-P_ | 30 × 30 × 10 mm^3^ of the active area | Up to 4 cm depth with different rotating angles up to ~60° | No | ^18^  (2022) |
| **US-TENG_DF-B_**  (multilayer structure) | Charging implantable electronic devices | ~26 V and ~6 mW at 3.5 cm  (ex vivo) | 40 kHz,  0.2 W cm^−2^  100 V_P-P_ | 30 × 30 × 4 mm^3^ dimension of active area | Up to 6 cm depth and up to ~60° misalignment | Yes | This work  (2025) |
